# IFNγ Signaling Endows DCs with the Capacity to Control Type I Inflammation during Parasitic Infection through Promoting T-bet+ Regulatory T Cells

**DOI:** 10.1371/journal.ppat.1004635

**Published:** 2015-02-06

**Authors:** Hyang-Mi Lee, Anne Fleige, Ruth Forman, Sunglim Cho, Aly Azeem Khan, Ling-Li Lin, Duc T. Nguyen, Aisling O'Hara-Hall, Zhinan Yin, Christopher A. Hunter, Werner Muller, Li-Fan Lu

**Affiliations:** 1 Division of Biological Sciences, University of California, San Diego, La Jolla, California, United States of America; 2 Technical University Braunschweig, Braunschweig, Germany; 3 Faculty of Life Sciences, University of Manchester, Manchester, United Kingdom; 4 Department of Human Genetics, Institute for Genomics and Systems Biology, University of Chicago, Chicago, Illinois, United States of America; 5 Department of Pathobiology, School of Veterinary Medicine, University of Pennsylvania, Philadelphia, Pennsylvania, United States of America; 6 The First Affiliated Hospital, International Imunology Center, The Biomedical Translational Research Institute, Key Laboratory of Molecular Immunology and Antibody Engineering of Guangdong Province, Jinan University, Guangzhou, Guangdong, People's Republic of China,; 7 Moores Cancer Center, University of California, San Diego, La Jolla, California, United States of America; Southwestern Medical Center at Dallas, UNITED STATES

## Abstract

IFNγ signaling drives dendritic cells (DCs) to promote type I T cell (Th1) immunity. Here, we show that activation of DCs by IFNγ is equally crucial for the differentiation of a population of T-bet+ regulatory T (Treg) cells specialized to inhibit Th1 immune responses. Conditional deletion of IFNγ receptor in DCs but not in Treg cells resulted in a severe defect in this specific Treg cell subset, leading to exacerbated immune pathology during parasitic infections. Mechanistically, IFNγ-unresponsive DCs failed to produce sufficient amount of IL-27, a cytokine required for optimal T-bet induction in Treg cells. Thus, IFNγ signalling endows DCs with the ability to efficiently control a specific type of T cell immunity through promoting a corresponding Treg cell population.

## Author Summary

In order to mount a protective response against numerous and enormously diverse microbial pathogens, T cells are able to differentiate into functionally distinct helper T (Th) subsets. To control different types of T cell immunity in a given inflammatory setting, regulatory T (Treg) cells have emerged as a dedicated immune population crucial for the negative regulation of immune responses. Here, we show that IL-27 derived from IFNγ responding dendritic cells (DCs) is crucial to drive the differentiation of a specialized Th1-Treg cell subset to limit IFNγ-mediated Th1 inflammation in a parasitic infection setting. The new cellular and molecular insights of our study not only challenge the current idea of Th1-Treg cell differentiation but also provide evidence of a more complex immune response and regulation scenario highlighted by the Treg cell-extrinsic and DC-intrinsic role of IFNγ signaling in Treg cell-mediated regulation of Th1 immunity particularly during *Toxoplasma gondii* infection.

## Introduction

T cells protect against numerous and enormously diverse microbial pathogens by taking cues from the environment, expressing unique “master transcription factors”, and differentiating into functionally distinct helper T (Th) subsets [1]. Each Th subset secretes signature cytokines and expresses distinct chemokine receptors that are pivotal for establishing proper host defense. At the same time, potent Th responses can also lead to deleterious immune-mediated inflammation and tissue damage, and therefore require adequate controls. To maintain this fine balance, a specialized subset of T cells termed regulatory T (Treg) cells has emerged as dedicated negative regulators of immune responses [2]. In Treg cells, the transcription factor Foxp3 orchestrates a distinct transcriptional program that enables them to establish and maintain immunological tolerance to ‘self’ and regulate immune responses to pathogens, commensals and tumors [3–5].

Similar to the Th cells they regulate, Treg cells come in “different flavors” phenotypically and functionally [6]. Rather than implementing a universal hard-wired suppressor program to limit many different types of immune responses, Treg cells employ distinct suppressor mechanisms that prominently feature in specific inflammatory and environmental settings. It has been revealed that the transcriptional machineries guiding the differentiation of conventional Th subsets in particular tissue environments can be utilized by Treg cells to efficiently control the corresponding classes of T cell immunity [7–11]. To date, the most extensively studied Treg cell subset has been a population of T-bet^+^ Treg cells that specialize in regulating type I inflammation [7,12,13]. The expression of T-bet, a Th1 cell lineage-specific transcription factor, confers competitive fitness, suppressor function and migration capacity to this Treg subset. Upregulation of CXCR3 facilitates their homing to the sites of Th1 inflammation to limit IFNγ-mediated immunopathology [7]. Mechanistically, it was shown that Stat1 activation by effector T cell-derived IFNγ in Treg cells was required for the differentiation of this so-called “Th1-Treg” cell subset [7,12]. Another recent study suggested that under different inflammatory conditions and in certain anatomical locations, IL-27, another cytokine that activates Stat1, plays a major role in promoting Th1-Treg cells [13]. Together, these results implied a complex scenario where multiple cellular and molecular factors are involved in the development of T-bet^+^ Treg cells and their regulation of Th1 inflammation.

In this study, we used a novel mouse model carrying a conditional allele of the signalling subunit of the IFNγ receptor (IFNγR2) to examine both Treg cell-intrinsic and extrinsic roles of IFNγ signaling in the development of Th1-Tregs. Our cell-specific type-restricted IFNγR2 ablation experiments revealed that while IFNγ signaling in Treg cells does not seem to play a significant role in promoting T-bet^+^ Treg cells, IFNγ responsiveness in CD11c^+^ dendritic cells (DCs) is critical for the development of this Th1-Treg cell population in both physiological and pathological settings. DCs unable to respond to IFNγ produced much reduced IL-27, leading to impaired T-bet induction in Treg cells. Consequently, mice with DC-specific IFNγR2 ablation harbored diminished numbers of T-bet^+^ Th1-Treg cells and suffered severe infection-induced Th1 immune pathology.

## Results

### IFNγ signaling in Treg cells is dispensable for Th1-Treg cell differentiation

**Fig 1:**
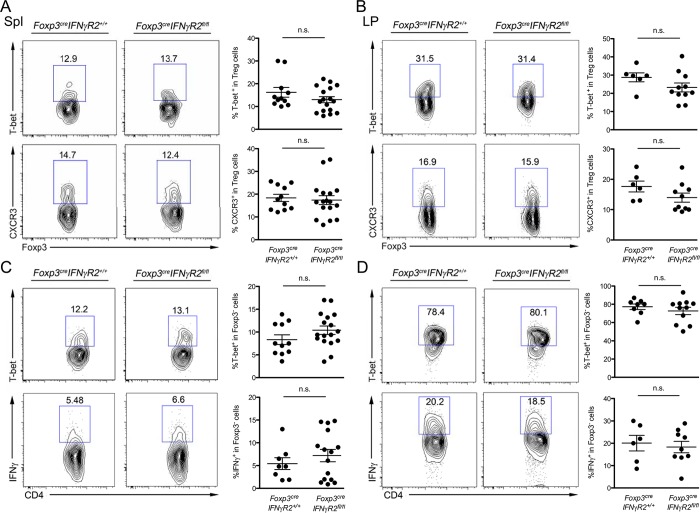
Minimal role of Treg cell-intrinsic IFNγ signaling in promoting T-bet^+^CXCR3^+^ Treg cells. FACS analysis and frequencies of T-bet^+^ or CXCR3^+^ cells in Foxp3^+^CD4^+^ Treg cells in **(A)** the spleen (Spl) and **(B)** the lamina propria (LP) of small intestine. FACS analysis and frequencies of T-bet^+^ or IFNγ^+^ Foxp3^-^CD4^+^ Teff cells in **(C)** Spl and **(D)** LP isolated from *Foxp3* ^*cre*^ *IFNγR2* ^*fl/fl*^ or *Foxp3* ^*cre*^ *IFNγR2* ^*+/+*^ mice. FACS plots shown are representative of three independent experiments.

Previously, it was shown that T-bet^+^CXCR3^+^ Treg cells failed to develop in mice devoid of IFNγR1, the ligand-binding chain of the IFNγ receptor [7]. A subsequent study using mixed bone marrow (BM) chimeras confirmed that activation of Stat1 through the engagement of IFNγR on Treg cells with effector T cell-derived IFNγ was essential to promote such a Treg cell population [12]. While these studies revealed the importance of IFNγ signaling in the differentiation of T-bet^+^CXCR3^+^ Treg cell population, their reliance on BM transfers precluded stringent mechanistic analysis of its role in Treg cell biology. To confirm and further expand on the aforementioned studies, we generated mice harboring a conditional *IFNγR2* allele (S1A Fig. in S1 Text). IFNγR2 is the non-ligand-binding chain of the IFNγ receptor essential for activating downstream signaling cascade upon IFNγ stimulation [14]. These mice were bred to mice with Foxp3-specific expression of Cre recombinase to produce mice with Treg cell-specific deletion of functional IFNγR (*Foxp3* ^*cre*^ *IFNγR2* ^*fl/fl*^) (S1B Fig. in S1 Text). As shown in S1C Fig. in S1 Text, no Stat1 phosphorylation could be detected in IFNγR2-deficient Treg cells from *Foxp3* ^*cre*^ *IFNγR2* ^*fl/fl*^ mice after IFNγ treatment while Teff cells from the same mouse or Treg cells from wildtype (WT) littermate controls responded to IFNγ normally. Surprisingly, and inconsistent with a previous study [12], the frequencies of T-bet^+^ or CXCR3^+^ Treg cells were comparable between *Foxp3* ^*cre*^ *IFNγR2* ^*fl/fl*^ mice and their WT littermate controls (Fig. 1A and B). Consequently, no increase in IFNγ production or T-bet expression in Foxp3^-^CD4^+^ effector T (Teff) cells could be observed in *Foxp3* ^*cre*^ *IFNγR2* ^*fl/fl*^ mice and these mice did not develop any clinical signs of autoimmune or inflammatory diseases (Fig. 1C, D). The results suggested IFNγ signaling in Treg cells is dispensable for the development of T-bet^+^CXCR3^+^ Th1-Treg cells.

### IFNγ signaling in CD11c^+^ DCs, but not myeloid cells, is required for Th1-Treg cell differentiation

**Fig 2:**
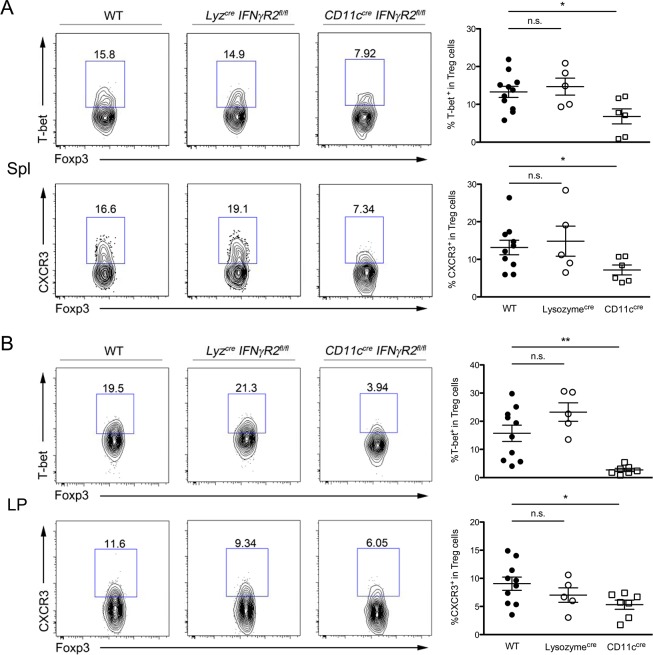
IFNγ signaling in CD11c^+^ DCs, but not myeloid cells, is required for the development of Th1-Treg cells. FACS analysis and frequencies of T-bet^+^ or CXCR3^+^ cells in Foxp3^+^CD4^+^ Treg cells in **(A)** Spl and **(B)** LP isolated from *CD11c* ^*cre*^ *IFNγR2* ^*fl/fl*^, *Lyz* ^*cre*^ *IFNγR2* ^*fl/fl*^ and WT control mice. FACS data are representative of four independent experiments (*p<0.05; **p<0.01).

Given that germline deletion of IFNγR1 or IFNγ in mice resulted in a significant loss of T-bet^+^CXCR3^+^ Treg cells, our unexpected new findings in *Foxp3* ^*cre*^ *IFNγR2* ^*fl/fl*^ mice suggested that IFNγ signaling through non-Treg cells were responsible for the development of the Th1-Treg subset [7,12]. To identify the major IFNγ responder cell subset(s) required for promoting Th1-Treg cells, we first induced DC- and myeloid cell-specific deletion of IFNγR2 by breeding *IFNγR2* ^*fl*^ mice to *CD11c* ^*cre*^ and *Lysozyme* ^*cre*^ (*Lyz* ^*cre*^) mice, respectively. Both populations can serve as antigen presenting cells (APCs) for Treg cells and the effects of IFNγ on their activation and function are well documented [15–17]. Analyses of *CD11c* ^*cre*^ *IFNγR2* ^*fl/fl*^ mice and *Lyz* ^*cre*^ *IFNγR2* ^*fl/fl*^ mice suggested that neither deletion of IFNγR2 in DCs nor in myeloid cells impacted the frequencies of total Foxp3^+^ Treg cells in both *CD11c* ^*cre*^ *IFNγR2* ^*fl/fl*^ and *Lyz* ^*cre*^ *IFNγR2* ^*fl/fl*^ mice (S2 Fig. in S1 Text). On the other hand, *CD11c* ^*cre*^ *IFNγR2* ^*fl/fl*^ mice showed reduced frequencies of T-bet^+^ or CXCR3^+^ Treg cells similar to what was reported in IFNγ-deficient mice [12], whereas *Lyz* ^*cre*^ *IFNγR2* ^*fl/fl*^ mice harbored equivalent numbers of T-bet^+^ and CXCR3^+^ Treg cells compared to WT controls (Fig. 2). These results indicated that CD11c^+^ DCs but not other myeloid cell populations function as the dominant immune cell subset responding to IFNγ to drive the development of Th1-Treg cell population.

### IFNγ unresponsiveness did not impact DC maturation and function at steady state

Next, we sought to examine whether reduced Th1-Treg cell numbers in *CD11c* ^*cre*^ *IFNγR2* ^*fl/fl*^ mice would lead to unrestrained IFNγ-mediated Th1 immune responses. Interestingly, both T-bet expression and IFNγ production from Foxp3^-^CD4^+^ Teff cells were similar if not lower in *CD11c* ^*cre*^ *IFNγR2* ^*fl/fl*^ mice compared to their WT littermates (S3 Fig. in S1 Text). The *CD11c* ^*cre*^ *IFNγR2* ^*fl/fl*^ mice remained healthy throughout the course of our study. These results seemed to argue against the notion that Th1-Treg cells are required to control Th1 immunity. However, it was also very possible that *CD11c* ^*cre*^ *IFNγR2* ^*fl/fl*^ mice lacked aberrant Th1 phenotype because DCs with impaired IFNγR signaling could not drive normal Th1 responses. Indeed, a recent study demonstrated that IFNγ-unresponsive DCs failed to produce sufficient amount of IL-12, a key cytokine required for the development and function of Th1 cells during Listeria infection, which led to impaired immune responses against the pathogen [18]. Likewise, reduced IL-12 production in IFNγR2 ablated DCs upon IFNγ stimulation could also be detected in our own study (S4 Fig. in S1 Text).

**Fig 3:**
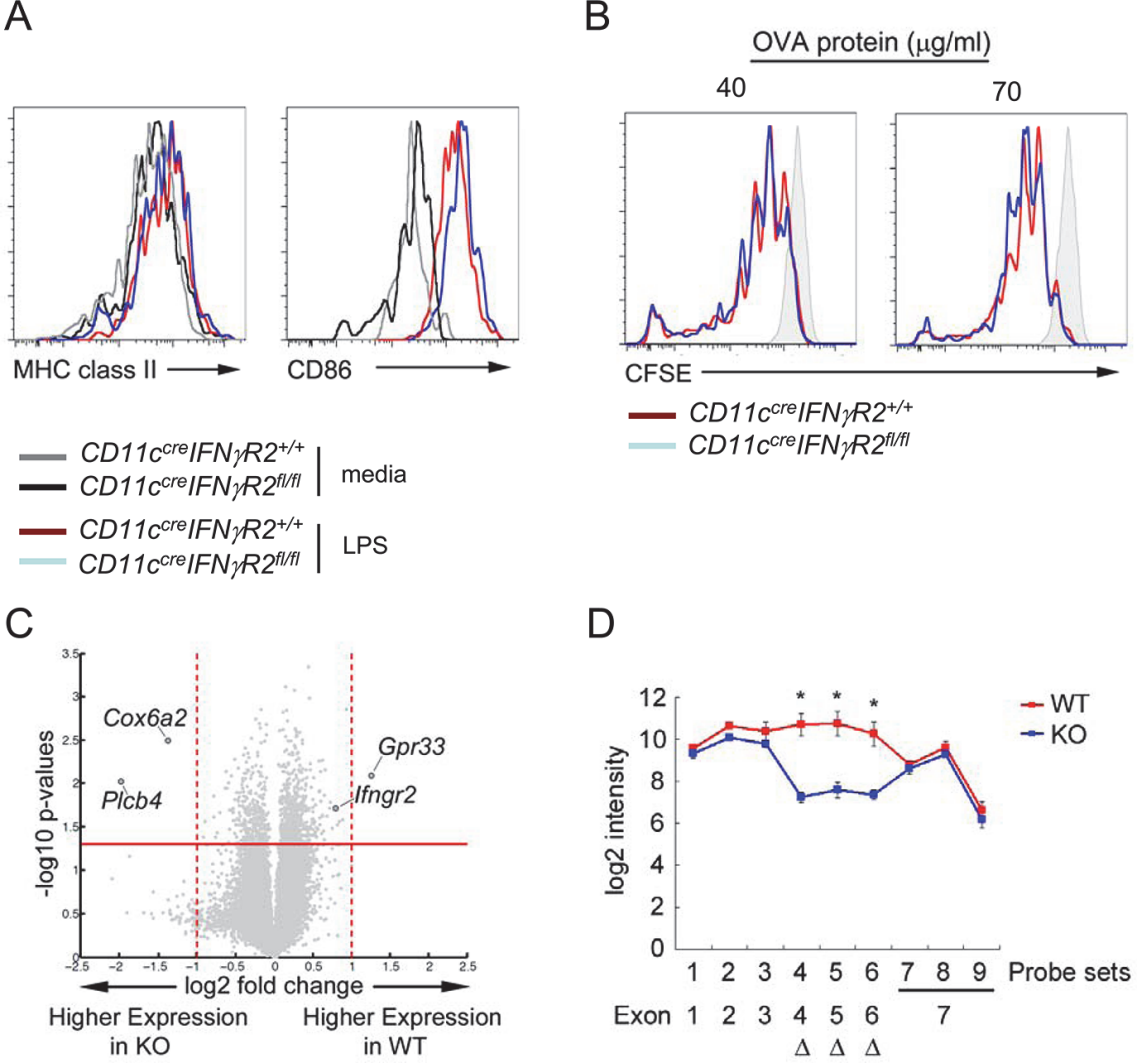
Dispensable role of IFNγR in DC maturation and function at steady state. FACS analysis of MHC class II and CD86 in **(A)** CD11c^+^ DCs with or without LPS stimulation for 24hr from *CD11c* ^*cre*^ *IFNγR2* ^*fl/f*^ and WT control mice. **(B)** Proliferation of OTII T cells co-cultured with DCs isolated from indicated mice pulsed with different does of OVA protein was shown by CFSE dilution. **(C)** Gene expression volcano plot, with—log 10 of the p value on the y axis and log 2 fold change on the x axis, such that genes with higher expression in WT DCs are on the right and genes with higher expression in KO DCs are on the left. **(D)** Signals (log2 intensity) of individual probe sets of *Ifngr2* gene and their locations on corresponding exons. All data are representative of three independent experiments. (*p<0.05).

This finding raised the question as to whether IFNγR2 ablation would broadly impact DC maturation and function or specifically impair the promotion of Th1-Treg cell differentiation. As mentioned previously, we did not observe any obvious difference in the frequencies and phenotypes of CD11c^+^ DCs from *CD11c* ^*cre*^ *IFNγR2* ^*fl/fl*^ mice. Nonetheless, we compared the expression levels of MHC class II and costimulation molecules in DCs isolated from *CD11c* ^*cre*^ *IFNγR2* ^*fl/fl*^ mice with WT controls in response to LPS stimulation. As shown in Fig. 3A, IFNγR2 ablation in DCs did not alter the expression of class II and CD86, and LPS stimulation resulted in a similar induction of both molecules (Fig. 3A). Next, we examined the ability of IFNγ-insensitive DCs to induce proliferation or differentiation of T cells. Both IFNγR2-sufficient and -deficient DCs pulsed with ovalbumin (OVA) protein induced equivalent proliferation of OTII CD4^+^ T cells that recognize an OVA peptide, indicating that ablation of IFNγR2 had minimal effect on the ability of DCs to process and present antigen and induce proliferation of T cells (Fig. 3B). To further corroborate these findings, we examined the impact of IFNγR2 deficiency on the gene expression profile of DCs. Consistent with our aforementioned *in vitro* studies, there was only a minimal difference between DCs isolated from *CD11c* ^*cre*^ *IFNγR2* ^*fl/fl*^ mice versus their WT counterparts (Fig. 3C). In fact, only three genes were identified with more than two-fold difference between IFNγR2-deficient and –sufficient DCs and neither gene has been demonstrated to exhibit an important function in immunity. As for *IFNγR2*, probe signals from three floxed and deleted exons (exon 4, 5, and 6; S1A Fig. in S1 Text) were more than 10-fold lower in IFNγR2-deficient DCs than those in WT samples while the probe signals located in other exons remained quite comparable between two groups (Fig. 3D). Such dilution effects resulted in a smaller difference in the expression of the whole *IFNγR2* gene (Fig. 3C). Taken together, our data suggested that IFNγR2 ablation did not significantly and globally impact the maturation and the function of DCs at steady state. Still, the lack of optimal IL-12 production in IFNγR2-deficient DCs upon IFNγ stimulation likely led to impaired Th1 responses, thereby masking the potential deleterious effects that might have resulted from the loss of Th1-Treg cells in *CD11c* ^*cre*^ *IFNγR2* ^*fl/fl*^ mice.

### Elevated IFNγ responses and exacerbated pathology in mice harboring IFNγR2-deficient DCs during *T*. *gondii* infection

T-bet^+^ Th1-Treg cells have been shown to be critical in limiting Th1 inflammation in many different autoimmune and infection settings [7,12,13]. While we could not detect at steady state a clear Th1 phenotype in *CD11c* ^*cre*^ *IFNγR2* ^*fl/fl*^ mice with reduced Th1-Treg cells, it was possible that the functional consequence of losing this cell population could be revealed in a strongly Th1-polarized environment when a robust IFNγ response occurs. To this end, we employed a model of *T*. *gondii* infection for which the role of Treg cells in controlling IFNγ-mediated Th1 inflammation has been well established [13,19]. Consistent with our observation in uninfected mice, despite having similar frequencies of total Foxp3^+^ Treg cells (S5A Fig. in S1 Text), infected *CD11c* ^*cre*^ *IFNγR2* ^*fl/fl*^ mice harbored significant reduced T-bet^+^ Treg cells compared to their WT counterparts in the Lamina Propria (LP) of the small intestine 4 days after infection (S5B Fig. in S1 Text). Moreover, comparable (or slightly increased) numbers of IFNγ-secreting Th1 Teff cells could be detected in mice harboring IFNγ-insensitive DCs (S5B Fig. in S1 Text), suggesting that IFNγ signaling in DCs does not play a non-redundant role in the generation of effector Th1 cell response during early *T*. *gondii* infection. Nonetheless, at this time point all mice remained largely symptom-free and no clear difference could be observed in mice regardless the presence or absence of IFNγR in DCs.

**Fig 4:**
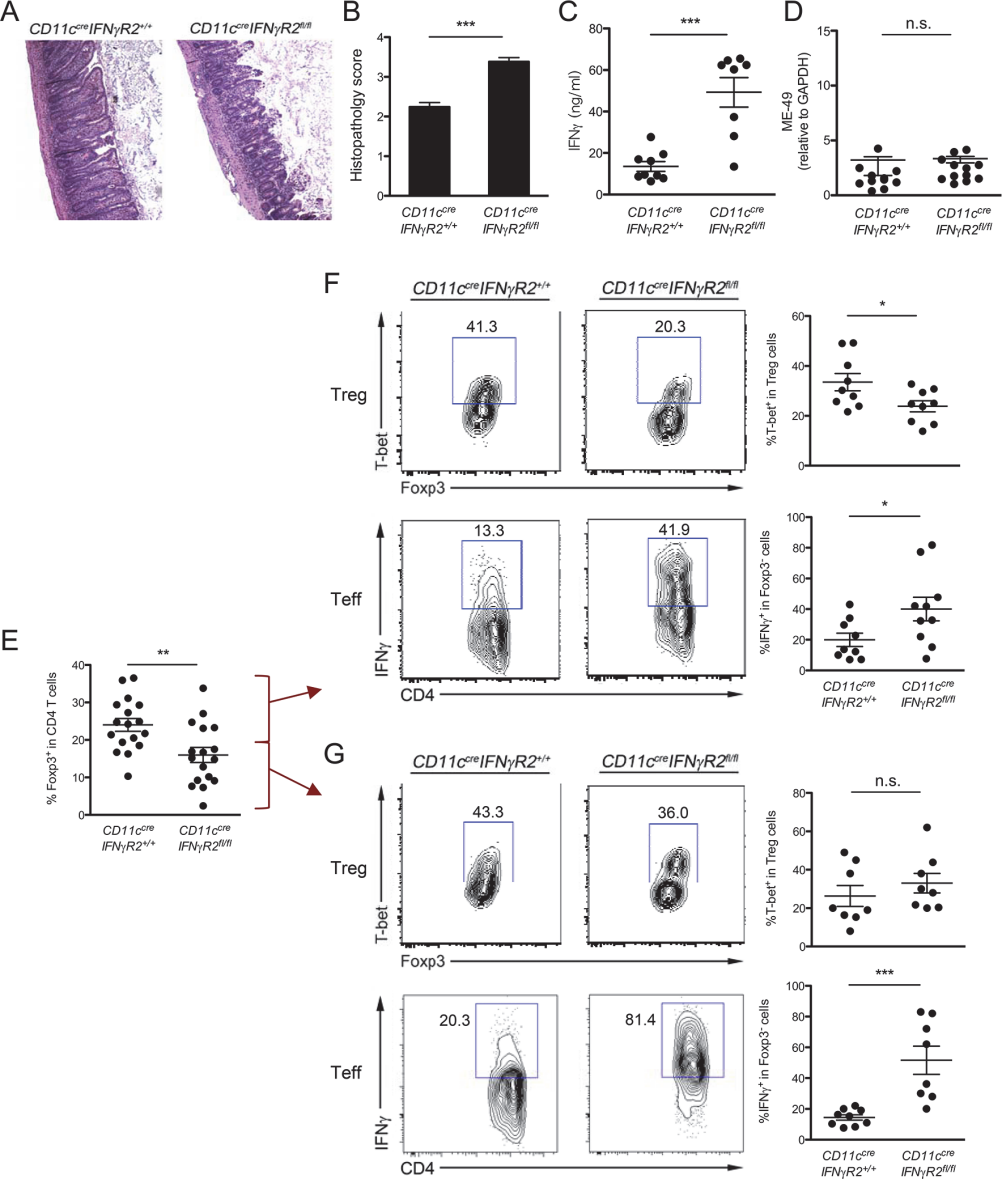
Reduced T-bet^+^ Th1-Treg cells in *CD11c* ^*cre*^ *IFNγR2* ^*fl/fl*^ mice resulted in unrestrained IFNγ-mediated Th1 inflammation during *T*. *gondii* infection. **(A,B)** Histological assessment of ileum from infected *CD11c* ^*cre*^ *IFNγR2* ^*fl/fl*^ and WT control mice (n = 12). **(C)** ELISA analysis of serum IFNγ levels and **(D)** PCR analysis of parasite burden in LP at days 8 after infection. **(E)** Frequencies of total Foxp3^+^ Treg cells from LP in *CD11c* ^*cre*^ *IFNγR2* ^*fl/fl*^ and WT control mice at day 8 post *T*. *gondii* infection. FACS analysis and frequencies of T-bet^+^ cells in Foxp3^+^CD4^+^ Treg cells and IFNγ^+^ cells in Foxp3^-^CD4^+^ Teff cells from LP in *CD11c* ^*cre*^ *IFNγR2* ^*fl/fl*^ **(F)** without or **(G)** with Treg cell collapse and their corresponding WT control mice at day 8 post *T*. *gondii* infection. FACS data are representative of three to four independent experiments. (*p<0.05; **p<0.01; ***p<0.001).

Next, to gain further insight into the potential role of Th1-Treg cells in limiting IFNγ-mediated Th1 inflammation, mice were examined 8 days after *T*. *gondii* infection when intestinal pathology could be observed [20].http://www.nature.com/mi/journal/v2/n6/full/mi2009105a.html - bib1 As shown in Fig. 4A and B, Day 8 infected-*CD11c* ^*cre*^ *IFNγR2* ^*fl/fl*^ mice exhibited more severe pathology in the small intestine including augmented thicken villi, greater loss of epithelial architecture in the ileum and the jejunum, massive inflammatory cell infiltration, and marked increased necrosis in Peyer’s patches compared to their WT littermates. It must be noted that the aforementioned phenotypes in *CD11c* ^*cre*^ *IFNγR2* ^*fl/fl*^ mice were most likely due to dysregulated IFNγ-mediated Th1 inflammation rather than uncontrolled parasite expansion, as a much higher titer of serum IFNγ could be easily detected in infected *CD11c* ^*cre*^ *IFNγR2* ^*fl/fl*^ mice whereas both *CD11c* ^*cre*^ *IFNγR2* ^*fl/fl*^ and WT control mice exhibited similar parasite burdens (Fig. 4C and D). Moreover, those *CD11c* ^*cre*^ *IFNγR2* ^*fl/fl*^ mice that suffered more severe intestinal pathology showed reduced overall Treg cell frequencies (Fig. 4E). Previously, it has been demonstrated that during lethal *T*. *gondii* infection, highly Th1 cell-polarized mucosal immune responses would result in the collapse of Treg cells [19]. To further confirm the observed phenotype of elevated dysregulated IFNγ responses in infected *CD11c* ^*cre*^ *IFNγR2* ^*fl/fl*^ mice was due to the selective impairment in Th1-Treg cell subset rather than the reduction of the entire Treg cell population, we first analyzed the group of *CD11c* ^*cre*^ *IFNγR2* ^*fl/fl*^ mice which retained similar frequencies of Treg cells as found in their corresponding WT littermates at day 8 post infection. Consistent with the proposed role of Th1-Treg cells in regulating IFNγ responses, reduced frequencies of T-bet^+^ Th1-Treg cells in *CD11c* ^*cre*^ *IFNγR2* ^*fl/fl*^ mice was accompanied by significant increases in IFNγ secreting Th1 Teff cells (Fig. 4F). Interestingly, when we analyzed the group of *CD11c* ^*cre*^ *IFNγR2* ^*fl/fl*^ mice in which the entire Treg cell population had collapsed, we were no longer able to observe any difference in the frequencies of T-bet^+^ Treg cells between *CD11c* ^*cre*^ *IFNγR2* ^*fl/fl*^ mice and littermate controls despite an even higher proportion of IFNγ-producing Teff cells in *CD11c* ^*cre*^ *IFNγR2* ^*fl/fl*^ mice (Fig. 4G). While these results were surprising, they were not entirely unexpected. Previous studies have demonstrated that Treg cells can acquire the effector Th1 cell phenotypes such as the upregulation of T-bet and the ability to produce IFNγ under extreme Th1-polarized conditions or in the absence of cell-intrinsic negative regulators of the Th1 cytokine signaling pathway (ie. miR-146a or SOCS1) [19,21]. As a consequence, these Treg cells with aberrant expression and activation of corresponding effector transcription factors promote inflammatory responses rather than suppression [22]. Consistent with this notion, a significant proportion of Treg cells from infected *CD11c* ^*cre*^ *IFNγR2* ^*fl/fl*^ mice with Treg cell collapse acquired the capacity of producing IFNγ contrary to what was observed in Treg cells from WT littermates or *CD11c* ^*cre*^ *IFNγR2* ^*fl/fl*^ mice harboring normal Treg cell frequencies (S6 Fig. in S1 Text). These results suggested that reduced Th1-Treg cells in mice with DCs incapable of responding to IFNγ would lead to the onset of the dyesregulated IFNγ-mediated inflammation during *T*. *gondii* infection. This Th1-Treg dependent immune pathology would likely have been exacerbated by further decrease in total Treg cell numbers and their acquisition of effector Th1 cell properties as by-stander effects of potent but poorly controlled Th1-driven inflammatory environments. In contrast, *Foxp3* ^*cre*^ *IFNγR2* ^*fl/fl*^ mice still exhibited frequencies of T-bet^+^ Th1-Treg cells similar to their WT littermates even under the heavily Th1-polarized condition during *T*. *gondii* infection (S7A Fig. in S1 Text), findings that were consistent with what was observed at steady state. As such, *Foxp3* ^*cre*^ *IFNγR2* ^*fl/fl*^ mice did not exhibit further IFNγ dysregulation compared to their WT littermates (S7B Fig. in S1 Text). Together, these results further demonstrated that IFNγ signaling in DCs but not in Treg cells is critical for promoting optimal Th1-Treg cells to control Th1 inflammation and associated immune pathology during *T*. *gondii* infection.

### IFNγ unresponsive DCs failed to upregulate IL-27 and other molecules with potential roles in Th1-Treg differentiation during *T*. *gondii* infection

**Fig 5:**
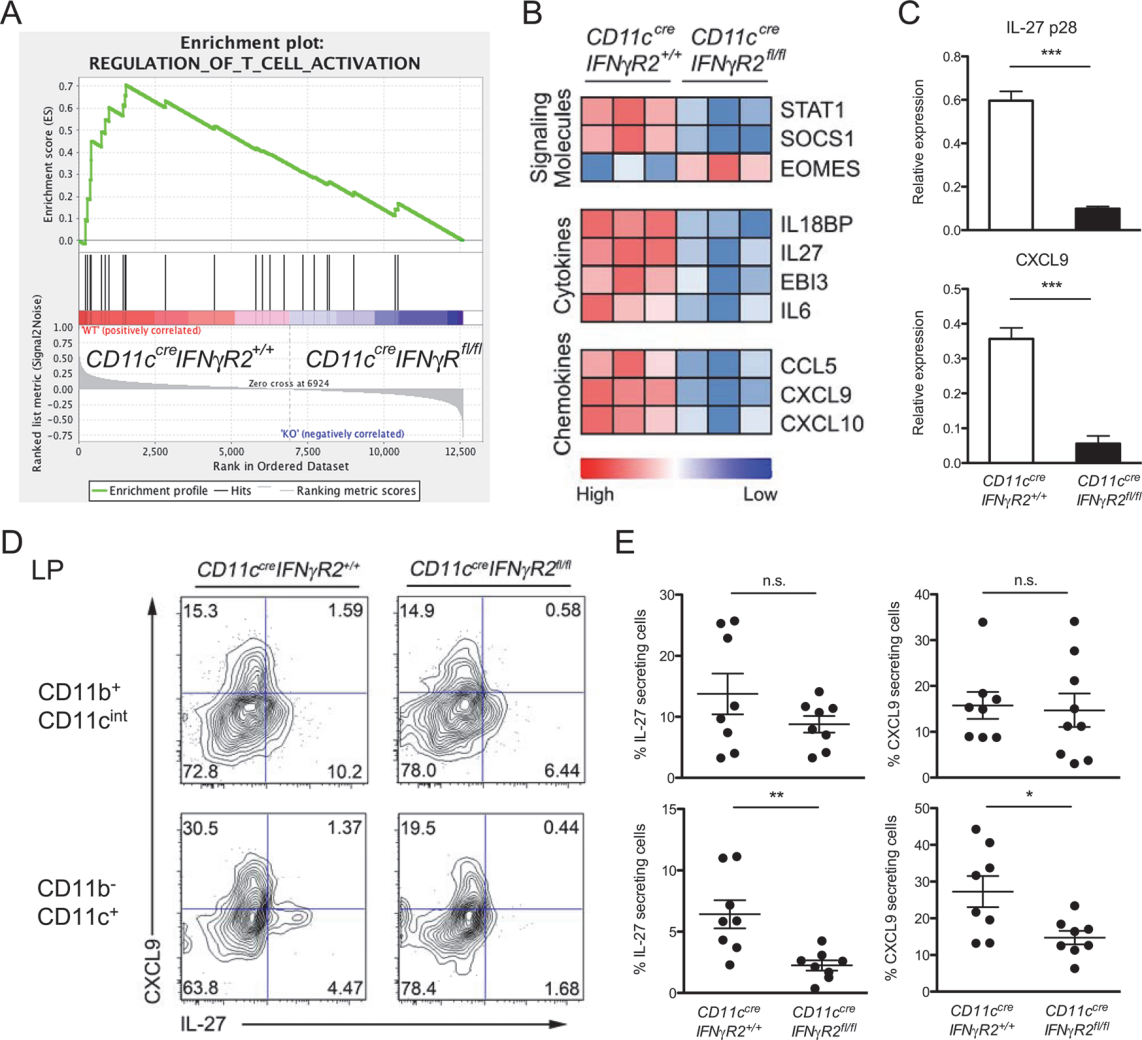
IFNγR2-deficient DCs failed to produce IL-27 as well as other molecules potentially important for Th1-Treg cell differentiation during *T*. *gondii* infection. **(A)** GSEA of T cell activation pathway genes (GO:0050863) between IFNγR2-deficient and—sufficient DCs [normalized enrichment score (NES) = 1.7312976, nominal P = 0.0, false discovery rate (FDR) < 25%] 8 days post *T*. *gondii* infection. **(B)** Comparison of genes with significant difference (p < 0.05) is shown of normalized expression values of candidate Th1-associated genes. Data are row normalized and presented as a heat map. **(C)** Expressions of IL-27(p28) and CXCL9 are further confirmed and quantified by qRT-PCR. **(D)** FACS analysis and **(E)** frequencies of CXCL9^+^ or IL-27^+^ CD11b^+^CD11c^int^ or CD11b^-^CD11c^+^ cells in LP isolated from indicated mice 8 days post *T gondii* infection. Data are representative of three independent experiments (*p<0.05; **p<0.01; ***p<0.001).

Thus far, we had demonstrated that IFNγR2 in DCs but not in Treg cells was essential for the development of Th1-Treg cells to limit Th1 inflammation during *T*. *gondii* infection. However, it remained unclear as to how IFNγ signaling could endow DCs with the capacity to promote Th1-Treg cells. To explore the molecular basis and to further identify the cell-intrinsic impact of IFNγ signaling in DCs during *T*. *gondii* infection, we used mixed BM chimeras approaches to directly compare the transcriptional responses in DCs with or without the capacity to respond to IFNγ during *T*. *gondii* infection (S8A Fig. in S1 Text). In brief, we transferred BM cells from *CD11c* ^*cre*^ *IFNγR2* ^*fl/fl*^ mice mixed with BM from congenically marked WT mice at a 1:1 ratio into Rag1-deficient recipients. Eight weeks after BM reconstitution, the resulting chimeric mice were orally infected with 40 cysts of *T*. *gondii*. Eight days after infection, IFNγR2-deficient and –sufficient DCs from the same chimeric mice were isolated and subjected to gene expression profiling analysis. Unlike that of DCs isolated from uninfected mice, a comparison of *CD11c* ^*cre*^ *IFNγR2* ^*fl/fl*^ and WT DCs isolated from *T*. *gondii* infected mice showed that more genes were differentially regulated (total of 153 genes >2 fold change; p<0.05) (S8B–S8D Fig. in S1 Text). Gene set enrichment analysis (GSEA) revealed significant enrichment of genes involved in the regulation of T cell activation differentially expressed between IFNγR2-deficient and –sufficient DCs, supporting the aforementioned roles of IFNγ-dependent regulation of T cell activation [23,24] (Fig. 5A). Additional screening of genes associated with Th1 immune responses revealed that *Stat1*, *Socs1*, *Il18bp*, *Il27(p28)*, *Ebi3*, *Il6*, *Ccl5*, *Cxcl9* and *Cxcl10* were downregulated and *Eomes* was upregulated in DCs incapable of responding to IFNγ (Fig. 5B).

Among the genes differentially regulated in IFNγ-unresponsive DCs, IL-27 was of particular interest. Our gene expression profiling analysis showed both IL-27 subunits, IL-27p28 and Ebi3, were downregulated in DCs incapable of responding to IFNγ (Fig. 5B). Similar to IFNγ, IL-27 signals through Stat1 and was recently shown to be able to induce T-bet^+^CXCR3^+^ Th1-Treg cell differentiation [13]. Mice devoid of IL-27 failed to develop Th1-Treg cells and consequently succumbed to lethal Th1 immune pathology during *T*. *gondii* infection [13]. It has been previously demonstrated that DCs produce high amounts of IL-27 upon *T*. *gondii* infection [13,19]. We, too, detected that *T*. *gondii* infected mice exhibited reduced gene and protein expressions of IL-27 in IFNγR2-deficient DCs compared to their WT counterparts, confirming the array results as well as the role of IFNγ signaling in driving the production of IL-27 in DCs (Fig. 5C and D). In contrast, the IL-27 levels in CD11b^+^CD11c^int^ cell population (i.e. monocytes and macrophage) remained largely unaltered in *CD11c* ^*cre*^ *IFNγR2* ^*fl/fl*^ mice and were most likely due to minor IFNγR2 deletion in these cells with relatively low levels of CD11c expression [13] (S9 Fig. in S1 Text).

Previously, several studies have demonstrated that IL-27 plays an important role in promoting the production of anti-inflammatory cytokine IL-10 in T cells [25–27]. Therefore, it was plausible that unrestrained Th1 inflammation in *CD11c* ^*cre*^ *IFNγR2* ^*fl/fl*^ mice during *T*. *gondii* infection was due to impaired IL-10 production by conventional T cells in the absence of sufficient amount of IL-27 produced by IFNγ-insensitive DCs. To test this possibility, we measured IL-10 production in T cells isolated from LP in infected *CD11c* ^*cre*^ *IFNγR2* ^*fl/fl*^ mice. As shown in S10 Fig. in S1 Text, while T cells routinely produced elevated levels of IFNγ in mice harboring IFNγ-insensitive DCs, they retained the capacity to produce comparable (if not higher) amounts of IL-10 during *T*. *gondii* infection.

### IL-27 produced by DCs is required for optimal Th1-Treg cell development through stimulating IL-27 receptor on Treg cells

The fact that Stat1 deficiency in Treg cells resulted in a complete loss of Th1-Treg cell population and that IL-27 activates Stat1 raised an interesting possibility: IFNγ signaling through DCs promotes the secretion of IL-27, which activates a Stat1 signaling cascade in Treg cells, leading to the development of this specialized Treg cell subset [12,13]. To first confirm the Treg cell-intrinsic role of IL-27 signaling in Th1-Treg cell differentiation *in vivo*, a mixed BM chimeras approach was taken. Analysis of the chimeric animals (*IL-27Rα* ^*-/-*^ */Ly5*.*1* ^*+*^ B6) allowed us to discriminate between Treg cell-intrinsic and-extrinsic effects of IL-27Rα deficiency on Th1-Treg cell development. As shown in S11 Fig. in S1 Text, reduced frequencies of T-bet^+^ Treg cells derived from IL-27Rα KO donor compared to their WT counterparts could be detected in the LP and to a lesser degree in the spleen, with or without *T*. *gondii* infection. These results were consistent with a recent study demonstrating that IL-27 could promote Th1-Treg cell development preferentially at mucosal sites [13]. More importantly, our findings further suggested Treg cell-intrinsic IL-27 signaling is necessary for generating optimal Th1-Treg cells locally and systemically at both physiological and inflammatory settings.

**Fig 6:**
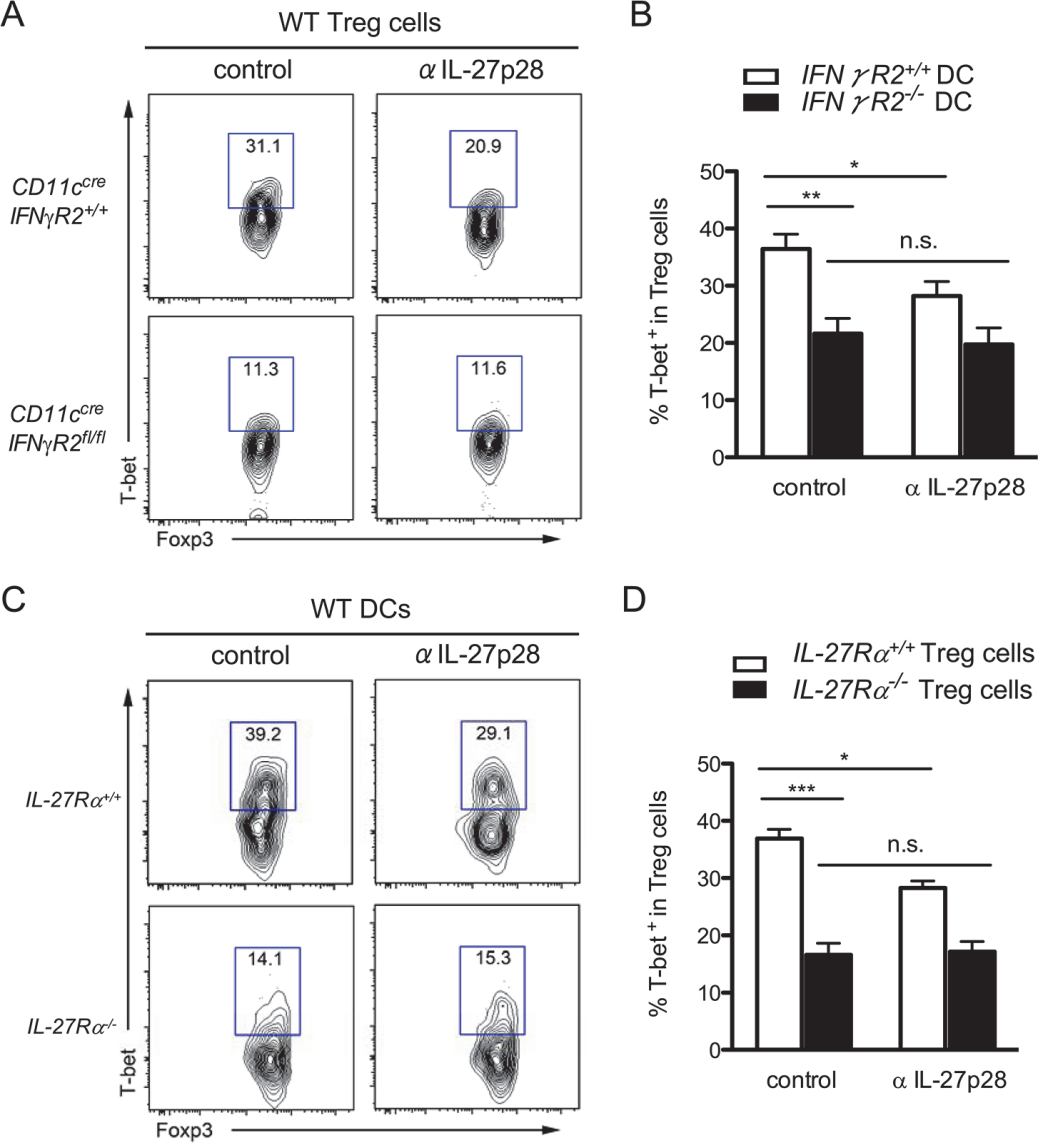
IL-27 secreted from *T gondii* infected-DC promotes T-bet^+^ Th1-Treg cell differentiation through stimulating IL-27R on Treg cells. **(A, B)** CD4^+^CXCR3^-^GFP^+^ Treg cells isolated from naive Foxp3^GFP^ mice were cultured with DCs from *CD11c* ^*cre*^ *IFNγR2* ^*fl/fl*^ or WT control mice day 6 post *T*. *gondii* infection. Isotype control or IL-27 neutralizing antibodies were added at the beginning of culture. **(C, D)** CD4^+^CXCR3^-^CD25^hi^ Treg cells isolated from naive IL-27Rα-deficient or WT control mice were cultured with DCs from WT mice day 6 post *T*. *gondii* infection in the presence of antibodies as indicated. FACS plots and histograms represent two or three independent experiments (*p<0.05; **p<0.01; ***p<0.001).

**Fig 7:**
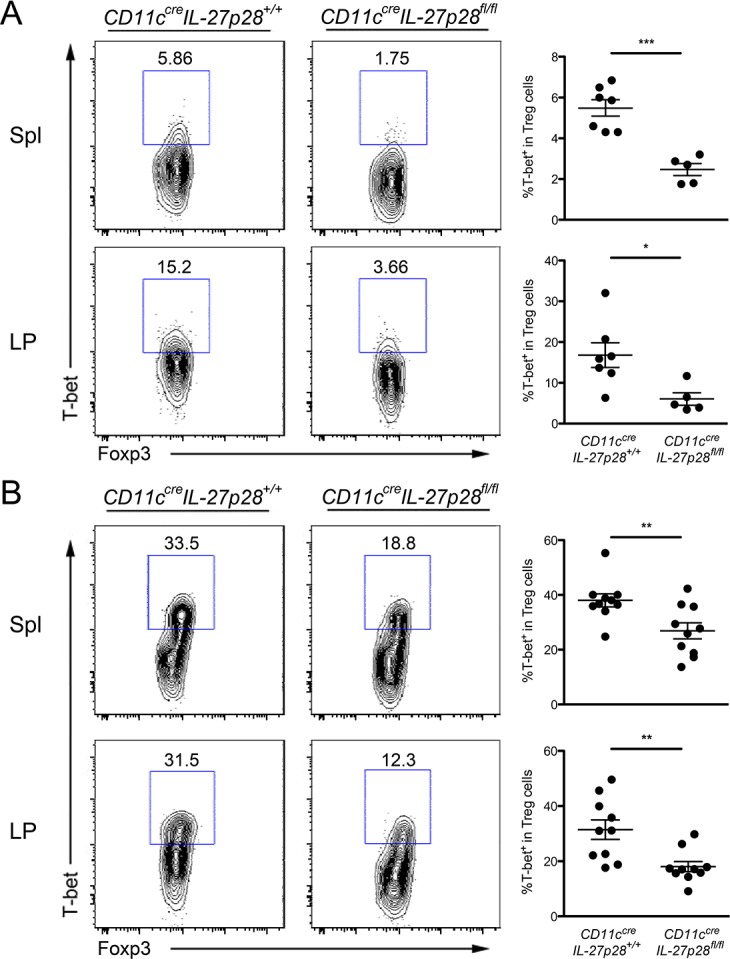
DC-derived IL-27 is critical for maintaining normal T-bet^+^ Th1-Treg cell population in both physiological and *T*. *gondii* infection settings. FACS analysis and frequencies of T-bet^+^ cells within Foxp3^+^CD4^+^ T cell population from spleen and LP in *CD11c* ^*cre*^ *IL27p28* ^*fl/fl*^ mice and WT littermate controls **(A)** at steady state or **(B)** 8 days after *T*. *gondii* infection. FACS plots are representative of three independent experiments (*p<0.05; **p<0.01; ***p<0.001).

Next we sought to determine whether IL-27 produced by DCs plays an indispensable role in DC-mediated T-bet^+^ Th1-Treg cell differentiation. It was previously demonstrated that DCs isolated from *T*. *gondii* infected mice were able to induce T-bet expression in Treg cells in an *in vitro* DC/Treg cell co-culture study [19]. Using the same method, we were able to confirm our *ex vivo* finding that IFNγR2-deficient DCs were less efficient at inducing T-bet expression in Foxp3^+^ Treg cells (Fig. 6A and B). More interestingly, neutralizing IL-27 also resulted in a significant albeit smaller reduction in T-bet^+^ Treg cells. As IL-27 blockade did not result in further reduced acquisition of T-bet by Treg cells cultured with IFNγR2-deficient DCs (Fig. 6A and B), this finding suggested that defective IL-27 production by IFNγ unresponsive DCs from infected mice contributes to the impaired Th1-Treg cell differentiation. Finally, IL-27Rα-deficient Treg cells also exhibited a more significant reduction in T-bet induction even in culture with WT DCs isolated from infected mice (Fig. 6C and D). The fact that blocking IL-27 did not further impact T-bet induction in IL-27Rα-deficient Treg cells suggested that IL-27 signaling in Treg cells but not in DCs was responsible for T-bet induction in Treg cells (Fig. 6C and D). Finally, to unequivocally demonstrate that DC-derived IL-27 is critical for the development of Th1-Treg cells *in vivo*, we examined mice with DC-specific ablation of IL-27 [28]. Consistent with our *in vitro* findings, significant reductions in T-bet^+^ Treg cell frequencies were observed in both spleen and LP of *CD11c* ^*cre*^ *IL27p28* ^*fl/fl*^ mice with or without *T*. *gondii* infection (Fig. 7A and B), pointing to DCs as critical cellular sources of IL-27 responsible for promoting the development and function of Th1-Treg cells in both physiological and parasitic infection settings.

## Discussion

DCs represent a critical component of the immune system that bridges innate and adaptive immunity [29]. Widely distributed in both lymphoid and non-lymphoid tissues, these sentinels alert the host to infection. During an infection, DCs are able to promote different types of Th responses pivotal for clearing a wide range of invading microbes [30]. At the same time, accumulating evidence has also demonstrated that DCs play a key role in establishing and maintaining peripheral tolerance through the induction and expansion of Treg cells [31]. Recently, it became evident that, like Th cells, Treg cells are able to differentiate into functionally distinct Treg cell subsets to control the corresponding type of T cell immunity [7–11]. As such, DCs could also play an integral part in orchestrating the differentiation of each of those specialized Treg cell populations. In this study, we demonstrated that IFNγ signaling specifically in DCs is required to promote a subset of T-bet^+^ Treg cells dedicated to controlling Th1 inflammation. Mice harboring IFNγ-unresponsive DCs failed to generate sufficient Th1-Treg cells, leading to much more severe immune pathology during *T*. *gondii* infection. The data presented here provides new cellular and molecular insights into Treg cell-mediated regulation of Th1 immunity, highlighted by the Treg cell-extrinsic and DC-intrinsic role of IFNγ signaling.

Evidence from a mixed BM chimeras study suggested that IFNγ signaling in Treg cells is critical for the development of the T-bet^+^ Th1-Treg cells [12]. In our attempts to further characterize the role of IFNγ in Th1-Treg cell biology, we unexpectedly found that IFNγ signaling was required in DCs but not in Treg cells to drive the differentiation of this specialized Treg cell population. The most probable explanation for the discrepancy between these two studies was in our respective methods. The previous study relied on mixed BM chimeras. In contrast, we employed a conditional gene targeting strategy that allowed us to assess the role of IFNγ signaling in a cell-specific manner. It has been recognized that the transient lymphopenia caused by irradiation and the competitive nature of mixed BM chimeras often lead to accelerated and enhanced phenotypes which might not be revealed in unperturbed mice with cell-specific gene deletion [21]. As such, while we did not detect any significant change in Th1-Treg cells in our *Foxp3* ^*cre*^ *IFNγR2* ^*fl/fl*^ mice even during *T*. *gondii* infection, it remains possible that under certain extreme conditions, IFNγ signaling in both DCs and Treg cells is required for generating optimal Th1-Treg cell subset to prevent deleterious immune-mediated pathology.

Our study of *T*. *gondii* infection has shown that mice harboring IFNγ-insensitive DCs exhibited dysregulated IFNγ responses and suffered exacerbated immune-mediated pathology. Considering the fact that DCs play a central role in both innate and adaptive immunity, one could argue that the aforementioned Th1 disease phenotypes observed in *T*. *gondii* infected *CD11c* ^*cre*^ *IFNγR2* ^*fl/fl*^ mice should not necessarily be attributed to impaired Th1-Treg cell-mediated Th1 regulation. Indeed, a role of IL-27 in promoting IL-10 production in T cells has been well established [25,26,27]. IL-27 can also play an anti-inflammatory role in restricting Th1 responses even in the absence of Treg cells during malaria infection [28,32,33]. Although reduced IL-27 production by IFNγ-insensitive DCs could lead to IFNγ-mediated Th1 inflammation in a Treg cell-independent manner, we think the impaired Th1-Treg cells significantly contribute to the pathology observed in our study for the following reasons.

First, while it remains to be further examined as to whether IL-27 derived from other cell populations plays a more critical or compensatory role in promoting the production of IL-10 by T cells, we did not detect a significant alteration in frequencies of IL-10-secreting T cells in *CD11c* ^*cre*^ *IFNγR2* ^*fl/fl*^ mice. Second, unlike what was described in the malaria study where Treg cells did not seem to play an important role in controlling Th1 responses [33], the protective role of Treg cells in restricting Th1 inflammation during *T*. *gondii* infection has been well recognized [19]. Moreover, while IL-27 could still directly suppress Th1 responses, the fact that IL-27-conditioned Treg cells were superior than unmanipulated Treg cells in rescuing IL-27-deficient mice from lethal immune pathology further supports the crucial role of IL-27-driven Th1-Treg cells in controlling immune responses during *T*. *gondii* infection [13]. Third, as the generation and function of effector Th1 cells did not seem altered in *CD11c* ^*cre*^ *IFNγR2* ^*fl/fl*^ mice during early *T*. *gondii* infection, it is unlikely that IFNγ unresponsiveness conferred enhanced Th1-inducing capacity to DCs that could have accounted for the dysregulated Th1 inflammation. Finally, despite a potential concern as to whether a protective anti-*Toxoplasma* immunity can develop in *CD11c* ^*cre*^ *IFNγR2* ^*fl/fl*^ mice, the exacerbated pathology in those mice did not seem to be parasite-driven. Comparable burdens of *T*. *gondii* were detected in both *CD11c* ^*cre*^ *IFNγR2* ^*fl/fl*^ mice and WT mice, suggesting that IFNγ-unresponsive DCs were still able to mount sufficient immune responses to control parasitic expansion. We believe our collective evidence proves that impaired T-bet^+^ Th1-Treg cell differentiation was the major contributor to the unrestrained Th1 inflammation observed in *CD11c* ^*cre*^ *IFNγR2* ^*fl/fl*^ mice during *T*. *gondii* infection. Furthermore, the highly inflammatory conditions produced by poorly regulated immune responses to parasitic infection can lead to the collapse of the entire Treg cell population and cause Foxp3^+^ cells to acquire effector Th1 cell properties, a compounding scenario which we observed in *CD11c* ^*cre*^ *IFNγR2* ^*fl/fl*^ mice with the most severe disease phenotypes.

The fact that we could not detect a significant Treg cell-intrinsic role of IFNγ signaling in the development of Th1-Treg cells in our cell-specific gene deletion study raised another interesting question as to whether Stat1 activation in Treg cells might also have been dispensable. After all, the Treg cell-intrinsic role of Stat1 activation in Th1-Treg cell differentiation was demonstrated by using a mixed BM chimeras approach similar to what was described in the previous IFNγR1 study [12]. A recent study of mice with T cell-specific Stat1 deletion demonstrated that Stat1 activity in T cells contributed to the development of IFNγ-producing Th1 cells and was required to generate a protective secondary anti-*Listeria* immunity in immunized animals [34]. Moreover, in mice with Treg cell-specific Stat1 ablation, almost no T-bet^+^CXCR3^+^ Treg cells could be detected. Consequently, these mice exhibited dysregulated IFNγ responses and developed exacerbated immune pathology, further supporting an irreplaceable Treg cell-intrinsic role of Stat1 activation in Th1-Treg cell differentiation (A. Chaudhry and A. Rudensky, personal communication).

Our findings strongly suggested that IL-27 serves as a functional link between IFNγ signaling in DCs and Stat1 activation in Treg cells during Th1-Treg cell development. Nonetheless, it remains possible that Stat1 activation by other DC-derived cytokines could also contribute to the development of Th1-Treg cell population. For example, IL-6, another cytokine that was identified in our transcriptional profiling study, is able to signals through both Stat3 and Stat1 in CD4 T cells [35]. It is plausible that loss of IL-6 production in IFNγ-insensitive DCs could also contribute to impaired Th1-Treg cells differentiation observed in *CD11c* ^*cre*^ *IFNγR2* ^*fl/fl*^ mice. Characterization of the T-bet^+^ Th1-Treg cell population from mice with DC-specific IL-6 deletion or with Treg cell-specific IL-6R (IL-6 receptor) ablation should provide further insights into the potential involvement of IL-6 signaling in Th1-Treg cell-mediated regulation of Th1 immunity particularly in an infection setting. Moreover, despite the opposite roles of Th1 and Th1-Treg cells during an inflammatory response, these two T cell populations share many properties. They display similar migration and homeostatic capacities necessary to carry out their respective functions in response to the same environmental cues. It is thus likely that other IFNγ-induced DC-derived immune mediators beside Stat1-activating cytokines could also play a role in Th1-Treg cell differentiation. For instance, DCs with impaired IFNγ signaling produced less CXCL9 and CXCL10, two chemokines which have both been shown to be required for Th1 cell differentiation through facilitating the interaction between DCs and CXCR3 expressing T cells *in vivo* [36]. Considering the fact that Th1-Treg cells express a high level of CXCR3 on their surface [12], it is possible that interaction between CXCR3-expressing Th1-Treg cells and CXCL9/CXCL10-expressing DCs is also needed to promote an optimal Th1-Treg cell population in an IFNγ-mediated Th1 inflammatory environment. Further genetic studies employing DC-specific ablation of CXCL9 and/or CXCL10 are required to directly test this idea.

In summary, our study of cell-type-specific IFNγR2 ablation has revealed an indispensable role of IFNγ signaling in DCs in the context of Treg cell-mediated regulation of Th1 immunity. Mechanistically, we have shown that IL-27 produced by DCs in response to IFNγ is critical to promote a subset of Treg cells that specialize in limiting Th1-driven inflammation. As a consequence of defective negative regulation, mice harboring DCs devoid of IFNγR2 exhibited unrestrained IFNγ responses and suffered exacerbated immune-mediated pathology during parasitic infections. Ultimately, our study has reinforced the notion that DCs function as a central player in controlling T cell-mediated immune responses and further demonstrated that DCs can integrate environmental cues to regulate the balance between tolerance and a specific type of immunity.

## Materials and Methods

### Ethics statement

This study was carried out in strict accordance with the recommendations in the Guide for the Care and Use of Laboratory Animals of the National Institutes of Health under a protocol (S11320) approved by the Institutional Animal Care and Use Committee at the University of California, San Diego (Animal Welfare Assurance Number: A3033–1). All efforts were made to minimize suffering of animals employed in this study.

### Mice

The targeting vector for *IFNγR2* was constructed using the RED/ET cloning system of gene bridges (http://www.genebridges.com). Briefly, a loxP flanked neomycin cassette and an xho1 site was inserted between exon 3 and exon 4 by ET cloning. Then an 8 kb long fragment of the *IFNγR2* gene, containing the region from exon 2 to exon 8 was subcloned into Bluescript SKII. The plasmid was then transfected into cre recombinase expressing bacteria. The neomycin resistance marker gene was removed, leaving one loxP site with an xho1 site in the modified gene. Subsequently a frt-flanked neomycin gene with one loxP site was inserted after exon 6. After verification, the targeting vector was transfected into IDF32F1 embryonic stem (ES) cells (C56BL/6 × 129S1), provided by Ralf Kühn from the Helmholtz centre, Munich. The ES cell clones were screened by southern blotting with a probe of exon 7 of the *IFNγR2* gene after xho1 restriction digest. One clone, F5, was selected and used to generate chimeric mice. Germline transmission was obtained and the FRT flanked neomycin gene was removed by breeding the mice to FLP deleter mice. The *IFNγR2* ^*fl*^ allele was functionally verified by crossing the allele to Cre deleter mice to generate mice carrying the *IFNγR2* KO allele (S1B Fig. in S1 Text). The *IFNγR2* ^*fl*^ mice were then backcrossed at least 8 times to C57BL/6J mice before crossing them to various cre recombinase driver lines, including *Foxp3* ^*cre*^ [37], *Lysozyme* ^*cre*^ and *CD11c* ^*cre*^ mice (from Jackson Laboratory). *IL27Rα* ^*-/-*^ mice were obtained from Dr. Elina Zuniga (University of California, San Diego) and *CD11c* ^*cre*^ *IL27p28* ^*fl/fl*^ mice were described previously [28].

### *T*. *gondii* Infection

The ME-49 strain of *T*. *gondii* was maintained in Swiss Webster and CBA/CaJ mice and tissue cysts from the brain were used for infection as previously described [13]. For all studies, 8- to 12-week old female mice were infected with 40 cysts of ME-49 by oral route and analyzed for Treg cell-mediated regulation of Th1 responses on day 8 post infection. To assess immunopathology, small intestines were removed and immediately fixed in 10% formalin solution for hematoxylin and eosin staining of sections embedded in paraffin. Inflammation was examined and scored by a UCSD pathologist on the following scale of 0–4: 0, within normal limits; 1, thickened villi and minimal inflammatory leukocyte infiltrates in the lamina propria; 2, some epithelial loss and mild to moderate inflammatory leukocytic and granulocytic infiltrates within the lamina propria and submucosa; 3, moderate to marked inflammatory infiltrates in the lamina propria, submucosa and muscularis externa; 4, marked to severe inflammation diffusely with altered or complete loss of normal histological structures and abundant inflammatory infiltrates that may extend into serosa.

To quantify parasite burden, real-time PCR was performed for DNA isolated from duodenum of infected mice using primers 5'-TCCCCTCtGCTGGCGAAAAGT-3' (forward) and 5'-AGCGTTCGTGGTCAACTATCGATTG-3' (reverse) to determine the relative abundance of *T*.*gondii B1* gene to mouse *Gapdh* gene. PCR reaction was run using the standard setting on the Applied Biosystems 7900 as described previously [38].

### Isolation of immune cells and flow cytometry

Single cell suspensions from the spleen were prepared by standard methods. Lamina propria lymphocytes were isolated as described elsewhere [13] with slight modification. Briefly, longitudinally cut small intestines were washed in cRPMI, and epithelial cells were removed (5mM EDTA and 1mM DTT), followed by digestion (0.16U/ml Liberase TL, Roche) and centrifugation with Percoll gradient to enrich lymphocytes. Cells were stained in FACS buffer (5% FBS in PBS) containing Fixable Viability Dye eFluor 780 or 450 (eBioscience) with the following antibodies for surface staining: CD4 and CXCR3 (all eBioscience). Intracellular staining of Foxp3, T-bet, IFNγ and IL-10 was done with Foxp3 staining buffer set according to manufacturer’s instructions (all eBioscience). To detect cytokine production, 2x10^6^ cells were stimulated in a 96 well plate with PMA, ionomycin and Brefeldin A solution for 4hr at 37°C before staining. Data was analyzed with FlowJo software (TreeStar).

### ELISA

Serum was collected from peripheral blood of mice 8 days after *T*. *gondii* infection and the serum IFNγ levels were quantified according to the manufacturer’s instructions (Biolegend).

### Quantitative RT-PCR

Total RNA was extracted from purified DCs by using RNeasy kits (Qiagen). For detecting expression of cytokines and chemokines, real-time PCR was performed using SYBR Green PCR kits (Applied Biosystems) for cDNAs generated by iScript^TM^ cDNA synthesis kit (Bio-Rad). Primers are as follows: IL-12 p40, 5'- AGGTCACACTGGACCAAAGG-3' (F) and 5'- TGGTTTGATGATGTCCCTGA-3' (R); IL-27 p28, 5'- CTCTGCTTCCTCGCTACCAC-3' (F) and 5'- AGGGGCAGCTTCTTTTCTTC-3' (R); CXCL9, 5'- TTTTCCTTTTGGGCATCATCTT-3' (F) and 5'- AGCATCGTGCATTCCTTATCACT-3' (R); CXCL10, 5'- GAAATCATCCCTGCGAGCCT-3' and 5'- TTGATGGTCTTAGATTCCGGATTC-3' (R); Gapdh, 5'- CGTCCCGTAGACAAAATGGT-3' (F) and 5'- TCAATGAAGGGGTCGTTGAT-3' (R).

### Analysis of DC functions

Splenic DCs were purified from *CD11c* ^*cre*^ *IFNγR2* ^*fl/fl*^ or WT mice by using CD11c beads (Miltenyi Biotech) with >90% purity. As readouts of maturation, purified DCs were stained for MHC class II and CD86 (all eBioscience) 24hr after LPS treatment (50ng/ml). In T cell proliferation assay, CFSE labeled OT II T cells (1x10^5^) were cultured with aforementioned DCs (4x10^4^) pulsed with OVA protein in 96 well plate for 60hr. For in vitro polarization assay, FACS sorted CD4^+^CD25^-^CD62L^hi^ naïve T cells (2.5x10^5^) were incubated with purified DCs (5x10^5^) for 4 days in the presence of IL-12(10U/ml) or IL-4 (20ng/ml) with anti-IFNγ (10μg/ml) and anti-IL-12 (10μg/ml) under Th1 or Th2 conditions, respectively. Cytokine production was assessed by flow cytometry with intracellular IFNγ and IL-4 staining.

### Generation of bone marrow chimeras

Mixed bone marrow chimeras were generated by transferring BM cells from *CD11c* ^*cre*^ *IFNγR2* ^*fl/fl*^ or *IL-27Rα* ^*-/-*^ mice and C57BL6 Ly5.1 mice as 1:1 ratio into lethally irradiated *Rag1* ^*-/-*^ recipients, as described previously [39].

### Gene expression profiling analysis

For microarray analysis, CD11c^+^ MHC class II^hi^ DCs from *CD11c* ^*cre*^ *IFNγR2* ^*fl/fl*^ or WT littermates or from mixed BM chimeras were sorted on FACSAria (Becton Dickinson). Isolated RNA was subject to gene expression profiling analysis using Mouse Gene 2.0 ST array (Affymetrix). Microarray samples were analyzed with R using bioconductor packages (affy, oligo, limma and pd.mogene.2.0.st). Probe level data was normalized using log2 average signal intensity and summarized to gene expression using median of probe values. Where indicated, replicates of each sample were grouped to calculate and cluster class means. Gene expression data was visualized using a volcano plot. Gene Set Enrichment Analysis (GSEA) of all GO biological processes (C5:BP) was performed using GSEA v4.0 (Broad Institute, M.I.T.). Microarray data are available from NCBI under accession number GSE64594.

### *In vitro* stimulation of Treg Cells by DCs

5x10^4^ CXCR3^low^CD4^+^CD25^hi^ or CXCR3^low^CD4^+^Foxp3GFP^+^ T cells isolated from naive *IL-27Rα* ^*-/-*^ mice or Foxp3^GFP^ mice were co-cultured in each well with CD11c^+^MHC class II^hi^ DCs from day 6 infected mice (ratio 1:1) with anti-CD3 mAb (1 μg/ml) in the presence or absence of anti-IL-27 (20 μg/ml). After a 2 day culture, T-bet, CXCR3 and Foxp3 staining was performed as previously described.

### Statistical analysis

Unpaired Student’s t test (or one way ANOVA tests for array analysis) was performed using Prism software (GraphPad). * p<0.05, ** p<0.01, and *** p<0.001 in all data.

## Supporting Information

S1 Text Contains S1-S11 Figs. **S1 Fig. Generation of mice harboring a conditional IFNγR2 allele. (A)** Schematic representation of the IFNγR2 targeting strategy. **(B)** Immunoblot analysis of IFNγR2 in naive Tconv (T_N_) and Treg (T_R_) cells isolated from *Foxp3* ^*cre*^ *IFNγR2* ^*fl/fl*^ and WT control mice. **(C)** FACS analysis of phosphorylation of Stat1 in Treg or Teff cells from in *Foxp3* ^*cre*^ *IFNγR2* ^*fl/fl*^ and WT control mice.in response to IFNγ stimulation. FACS data are representative of three independent experiments. **S2 Fig. No difference in total Treg cell numbers was observed in mice with DC- or myeloid cell-specific ablation of IFNγR2**. Frequencies of Foxp3^+^ Treg cells from spleen in *CD11c* ^*cre*^ *IFNγR2* ^*fl/fl*^, *Lyz* ^*cre*^ *IFNγR2* ^*fl/fl*^ or WT control mice. Data are representative of two experiments and each dot represents an individual mouse. **S3 Fig. Deletion of IFNγR in DCs does not lead to dysregulated IFNγ-mediated Th1 responses**. FACS analysis and frequencies of T-bet^+^ or IFNγ^+^ Foxp3^-^CD4^+^ T cells isolated from **(A)** spleen or **(B)** LP of small intestine in *CD11c* ^*cre*^ *IFNγR2* ^*fl/fl*^ or WT control mice. FACS data are representative of three independent experiments and each dot represents an individual mouse. **S4 Fig. IFNγ signaling in DCs is essential to drive the expression of IL-12. (A)** FACS and **(B)** qRT-PCR analysis of IL-12 expression in CD11c^+^ DCs isolated from *CD11c* ^*cre*^ *IFNγR2* ^*fl/fl*^ mice or WT control mice in response to IFNγ stimulation. Data are representative of two independent experiments. (*p<0.05). **S5 Fig. Comparable effector Th1 cell responses in mice harboring IFNγ-insensitive DCs during early phase of *T*. *gondii* infection. (A)** Frequencies of total Foxp3^+^ Treg cells and **(B)** FACS analysis and frequencies of T-bet^+^ cells in Foxp3^+^CD4^+^ Treg cells and IFNγ^+^ cells in Foxp3^-^CD4^+^ Teff cells from LP in *CD11c* ^*cre*^ *IFNγR2* ^*fl/fl*^ or WT control mice at days 4 after infection. FACS data are representative of two independent experiments and each dot represents an individual mouse. (**p<0.01). **S6 Fig. Acquisition of IFNγ-producing capacity by Treg cells from *CD11c*** ^***cre***^ ***IFNγR2*** ^***fl/fl***^ **mice with collapse in total Treg cell population during *T*. *gondii* infection**. FACS analysis and frequencies of IFNγ^+^ cells in Foxp3^+^CD4^+^ Treg cells from LP in WT control mice and *CD11c* ^*cre*^ *IFNγR2* ^*fl/fl*^ mice with or without Treg cell collapse at days 8 after infection. FACS data are representative of three to four independent experiments and each dot represents an individual mouse. (**p<0.01). **S7 Fig. Deletion of IFNγR in Treg cells did not lead to reduced Th1-Treg cell frequencies and dysregulated IFNγ-mediated Th1 responses during *T*. *gondii* infection. (A)** FACS analysis and frequencies of T-bet^+^Foxp3^+^CD4^+^ Treg cells and **(B)** FACS analysis and frequencies of IFNγ^+^Foxp3^-^CD4^+^ Teff cells isolated from spleen or LP of small intestine in *Foxp3* ^*cre*^ *IFNγR2* ^*fl/fl*^ or WT control mice at days 8 after infection. FACS data are representative of three independent experiments and each dot represents an individual mouse. **S8 Fig. Gene expression profiling analysis in IFNγ-unresponsive DCs isolated from *T*. *gondii* infected mice. (A)** Schematic of mixed BM chimeras with *T*. *gondii* infection. **(B)** Gene expression volcano plot, with—log 10 of the p value on the y axis and log 2 fold change on the x axis. **(C)** Hierarchical clustering and heat map analysis with genes that were differentially regulated 2-fold or greater and p < 0.05 were performed. **(D)** Top 20 genes that were either upregulated or downregulated were shown. **S9 Fig. Cell-type specific deletion of IFNγR2**. qRT-PCR analysis of IFNγR2 expression in CD11c^+^ DCs or CD11b^+^ myeloid cells in *CD11c* ^*cre*^ *IFNγR2* ^*fl/fl*^ mice, *Lyz* ^*cre*^ *IFNγR2* ^*fl/fl*^ mice or their corresponding WT littermates. Data are representative of two independent experiments. (***p<0.001). **S10 Fig. Impaired IL-27 production by IFNγ-insensitive DCs did not result in reduced IL-10 secretion by effector T cells during *T*. *gondii* infection. (A)** FACS analysis and **(B)** frequencies IL-10^+^ cells in Foxp3^-^CD4^+^ Teff cells isolated from *CD11c* ^*cre*^ *IFNγR2* ^*fl/fl*^ and WT control mice day 8 post *T*. *gondii* infection. FACS data are representative of two independent experiments (n = 5). **S11 Fig. Treg cell-intrinsic IL-27 signaling is essential to maintain normal T-bet** ^**+**^ **CXCR3** ^**+**^ **Treg cell population at both physiological and *T*. *gondii* infection settings**. FACS analysis and frequencies of T-bet^+^ cells within each donor-derived Foxp3^+^CD4^+^ T cell population from spleen and LP in *IL-27Rα* ^*-/-*^ */*Ly5.1 B6 mixed BM chimeras and control chimeric mice **(A)** at steady state or **(B)** 8 days after *T*. *gondii* infection. FACS plots are representative of three independent experiments. (*p<0.05; **p<0.01; ***p<0.001).
(PDF)
