# Correction: IFNγ Signaling Endows DCs with the Capacity to Control Type I Inflammation during Parasitic Infection through Promoting T-bet+ Regulatory T Cells

**DOI:** 10.1371/journal.ppat.1004741

**Published:** 2015-03-16

**Authors:** The PLOS Pathogens Staff

There are errors in the Funding section. The correct funding information is as follows: This work was supported by NIH grants AI089935 and AI108651 (L.-F.L). L.-F.L. is a Kimmel Scholar and a Hellman Fellow. H.-M.L is an Irvington Fellow of the Cancer Research Institute. The conditional IFNγR2 mutant mouse generation was supported by DFG (SFB 621 to W.M.) and MUGEN LSHG-CT-2005-005203. The initial functional characterization of IFNγR2 mutant mice was supported by the BBSRC PhD program at the University of Manchester. The aforementioned funders had no role in study design, data collection and analysis, decision to publish, or preparation of the manuscript.
