# Supplementary material for: IFNγ Signaling Endows DCs with the Capacity to Control Type I Inflammation during Parasitic Infection through Promoting T-bet+ Regulatory T Cells
Source: PLoS Pathog. 2015 Feb 6;11(2):e1004635. doi: 10.1371/journal.ppat.1004635 (PMC4450074; doi:10.1371/journal.ppat.1004635)
Supplement: S1 Text — S1 Fig. Generation of mice harboring a conditional IFNγR2 allele. (A) Schematic representation of the IFNγR2 targeting strategy. (B) Immunoblot analysis of IFNγR2 in naive Tconv (TN) and Treg (TR) cells isolated from Foxp3 cre IFNγR2 fl/fl and WT control mice. (C) FACS analysis of phosphorylation of Stat1 in Treg or Teff cells from in Foxp3 cre IFNγR2 fl/fl and WT control mice.in response to IFNγ stimulation. FACS data are representative of three independent experiments. S2 Fig. No difference in total Treg cell numbers was observed in mice with DC- or myeloid cell-specific ablation of IFNγR2. Frequencies of Foxp3+ Treg cells from spleen in CD11c cre IFNγR2 fl/fl, Lyz cre IFNγR2 fl/fl or WT control mice. Data are representative of two experiments and each dot represents an individual mouse. S3 Fig. Deletion of IFNγR in DCs does not lead to dysregulated IFNγ-mediated Th1 responses. FACS analysis and frequencies of T-bet+ or IFNγ+ Foxp3-CD4+ T cells isolated from (A) spleen or (B) LP of small intestine in CD11c cre IFNγR2 fl/fl or WT control mice. FACS data are representative of three independent experiments and each dot represents an individual mouse. S4 Fig. IFNγ signaling in DCs is essential to drive the expression of IL-12. (A) FACS and (B) qRT-PCR analysis of IL-12 expression in CD11c+ DCs isolated from CD11c cre IFNγR2 fl/fl mice or WT control mice in response to IFNγ stimulation. Data are representative of two independent experiments. (*p<0.05). S5 Fig. Comparable effector Th1 cell responses in mice harboring IFNγ-insensitive DCs during early phase of T. gondii infection. (A) Frequencies of total Foxp3+ Treg cells and (B) FACS analysis and frequencies of T-bet+ cells in Foxp3+CD4+ Treg cells and IFNγ+ cells in Foxp3-CD4+ Teff cells from LP in CD11c cre IFNγR2 fl/fl or WT control mice at days 4 after infection. FACS data are representative of two independent experiments and each dot represents an individual mouse. (**p<0.01). S6 Fig. Acquisition of IFNγ-producing [file ppat.1004635.s001.pdf]

Supporting Information Legends

Figure S1. Generation of mice harboring a conditional IFNγR2 allele. (A) Schematic representation of the IFNγR2 targeting strategy. **(B)** Immunoblot analysis of IFNγR2 in naive Tconv (T_N) and Treg (T_R) cells isolated from *Foxp3^{cre}IFNγR2$^{fl/fl}$* and WT control mice. **(C)** FACS analysis of phosphorylation of Stat1 in Treg or Teff cells from in *Foxp3^{cre}IFNγR2$^{fl/fl}$* and WT control mice.in response to IFNγ stimulation. FACS data are representative of three independent experiments.

Figure S2. No difference in total Treg cell numbers was observed in mice with DC- or myeloid cell-specific ablation of IFNγR2. Frequencies of Foxp3$^+$ Treg cells from spleen in *CD11c^{cre}IFNγR2$^{fl/fl}$*, *Lyz^{cre}IFNγR2$^{fl/fl}$* or WT control mice. Data are representative of two experiments and each dot represents an individual mouse.

Figure S3. Deletion of IFNγR in DCs does not lead to dysregulated IFNγ-mediated Th1 responses. FACS analysis and frequencies of T-bet$^+$ or IFNγ$^+$ Foxp3$^-$CD4$^+$ T cells isolated from **(A)** spleen or **(B)** LP of small intestine in *CD11c^{cre}IFNγR2$^{fl/fl}$* or WT control mice. FACS data are representative of three independent experiments and each dot represents an individual mouse.

Figure S4. IFNγ signaling in DCs is essential to drive the expression of IL-12. (A) FACS and **(B)** qRT-PCR analysis of IL-12 expression in CD11c$^+$ DCs isolated from *CD11c^{cre}IFNγR2$^{fl/fl}$* mice or WT control mice in response to IFNγ stimulation. Data are representative of two independent experiments. (*$p<0.05$)

Figure S5. Comparable effector Th1 cell responses in mice harboring IFNγ-insensitive DCs during early phase of *T. gondii* infection. (A) Frequencies of total Foxp3$^+$ Treg cells and

(B) FACS analysis and frequencies of T-bet$^+$ cells in Foxp3$^+$CD4$^+$ Treg cells and IFNγ$^+$ cells in Foxp3$^-$CD4$^+$ Teff cells from LP in *CD11c^{cre}IFNγR2$^{fl/fl}$* or WT control mice at days 4 after infection. FACS data are representative of two independent experiments and each dot represents an individual mouse. (**p<0.01).

Figure S6. Acquisition of IFNγ-producing capacity by Treg cells from *CD11c^{cre}IFNγR2$^{fl/fl}$* mice with collapse in total Treg cell population during *T. gondii* infection. FACS analysis and frequencies of IFNγ$^+$ cells in Foxp3$^+$CD4$^+$ Treg cells from LP in WT control mice and *CD11c^{cre}IFNγR2$^{fl/fl}$* mice with or without Treg cell collapse at days 8 after infection. FACS data are representative of three to four independent experiments and each dot represents an individual mouse. (**p<0.01).

Figure S7. Deletion of IFNγR in Treg cells did not lead to reduced Th1-Treg cell frequencies and dysregulated IFNγ-mediated Th1 responses during *T. gondii* infection. (A) FACS analysis and frequencies of T-bet$^+$Foxp3$^+$CD4$^+$ Treg cells and **(B)** FACS analysis and frequencies of IFNγ$^+$Foxp3$^-$CD4$^+$ Teff cells isolated from spleen or LP of small intestine in *Foxp3^{cre}IFNγR2$^{fl/fl}$* or WT control mice at days 8 after infection. FACS data are representative of three independent experiments and each dot represents an individual mouse.

Figure S8. Gene expression profiling analysis in IFNγ-unresponsive DCs isolated from *T. gondii* infected mice. (A) Schematic of mixed BM chimeras with *T. gondii* infection. **(B)** Gene expression volcano plot, with – log 10 of the p value on the y axis and log 2 fold change on the x axis. **(C)** Hierarchical clustering and heat map analysis with genes that were differentially regulated 2-fold or greater and $p < 0.05$ were performed. **(D)** Top 20 genes that were either upregulated or downregulated were shown.

Figure S9. Cell-type specific deletion of IFNγR2. qRT-PCR analysis of IFNγR2 expression in CD11c$^+$ DCs or CD11b$^+$ myeloid cells in *CD11c^{cre}IFNγR2$^{fl/fl}$* mice, *Lyz^{cre}IFNγR2$^{fl/fl}$* mice or their corresponding WT littermates. Data are representative of two independent experiments. (***p<0.001).

Figure S10. Impaired IL-27 production by IFNγ-insensitive DCs did not result in reduced IL-10 secretion by effector T cells during *T. gondii* infection. (A) FACS analysis and **(B)** frequencies IL-10$^+$ cells in Foxp3$^-$CD4$^+$ Teff cells isolated from *CD11c^{cre}IFNγR2$^{fl/fl}$* and WT control mice day 8 post *T. gondii* infection. FACS data are representative of two independent experiments (n=5).

Figure S11. Treg cell-intrinsic IL-27 signaling is essential to maintain normal T-bet$^+$CXCR3$^+$ Treg cell population at both physiological and *T. gondii* infection settings. FACS analysis and frequencies of T-bet$^+$ cells within each donor-derived Foxp3$^+$CD4$^+$ T cell population from spleen and LP in *IL-27Rα$^{-/-}$*/Ly5.1 B6 mixed BM chimeras and control chimeric mice **(A)** at steady state or **(B)** 8 days after *T. gondii* infection. FACS plots are representative of three independent experiments. (*p<0.05; **p<0.01; ***p<0.001).

Figure S1.

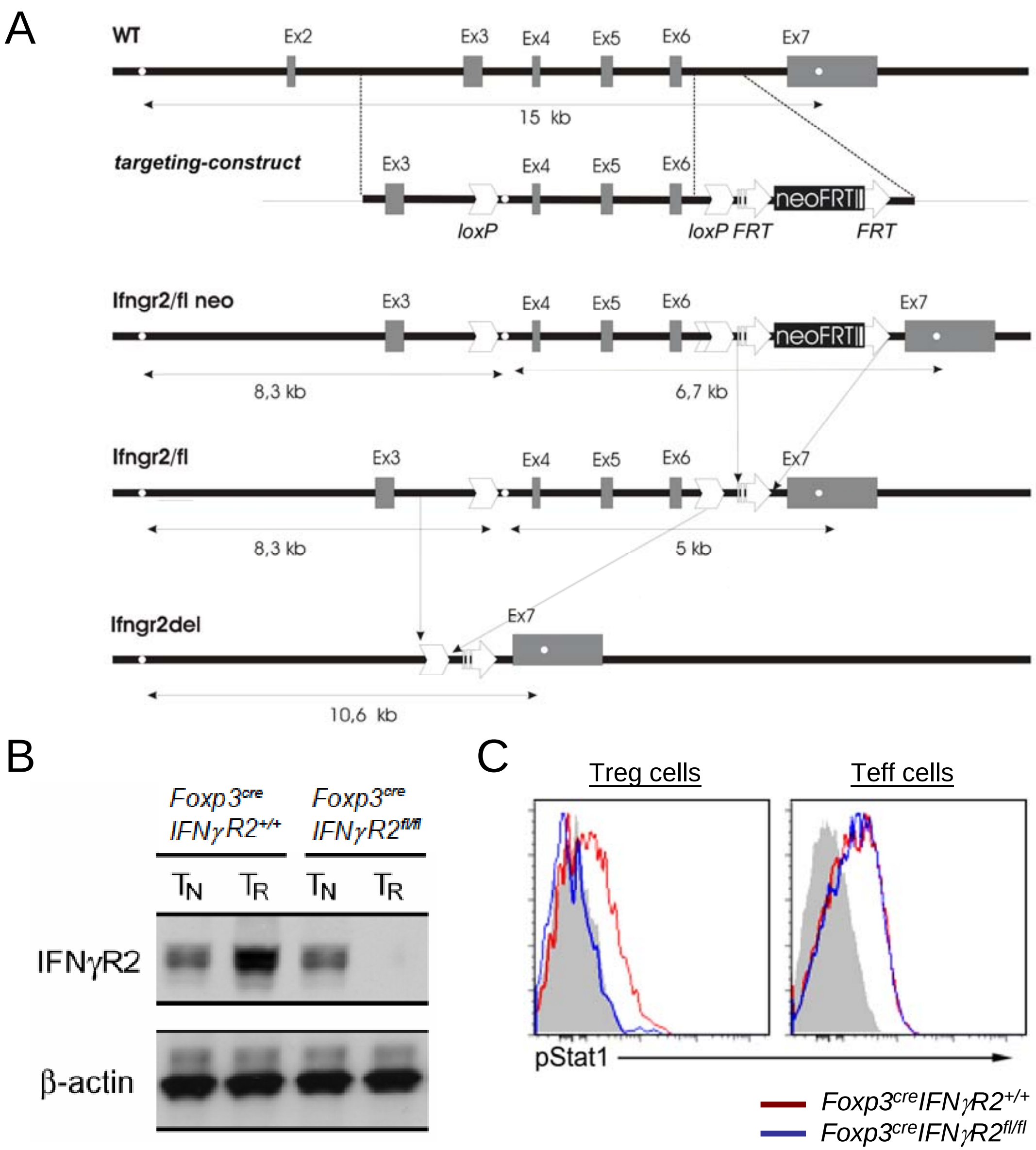

A
WT
Ex2
Ex3
Ex4
Ex5
Ex6
Ex7
15 kb
targeting-construct
loxP
loxP FRT
neoFRT
FRT
Ifngr2/fl neo
8,3 kb
6,7 kb
Ifngr2/fl
5 kb
Ifngr2del
10,6 kb
B
Foxp3cre IFNγR2+/+
Foxp3cre IFNγR2fl/fl
TN
TR
IFNγR2
β-actin
C
Treg cells
Teff cells
pStat1
Foxp3creIFNγR2+/+
Foxp3creIFNγR2fl/fl

Figure S2.

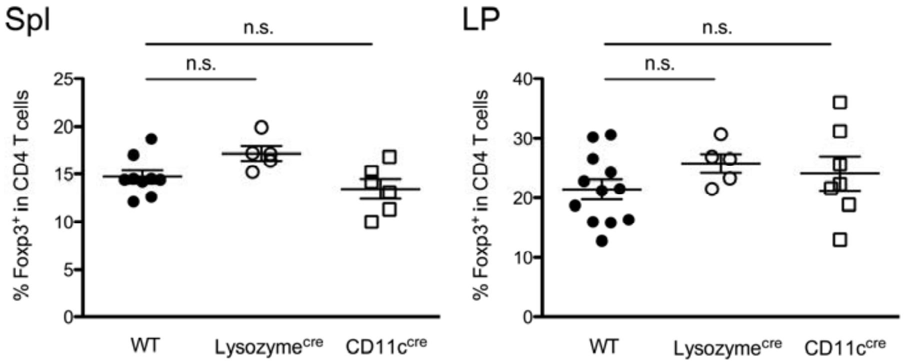

Spl
n.s.
n.s.
25
20
15
10
5
0
% Foxp3+ in CD4 T cells
WT
Lysozymecre
CD11ccre
LP
n.s.
n.s.
40
30
20
10
0
% Foxp3+ in CD4 T cells
WT
Lysozymecre
CD11ccre

Figure S3.

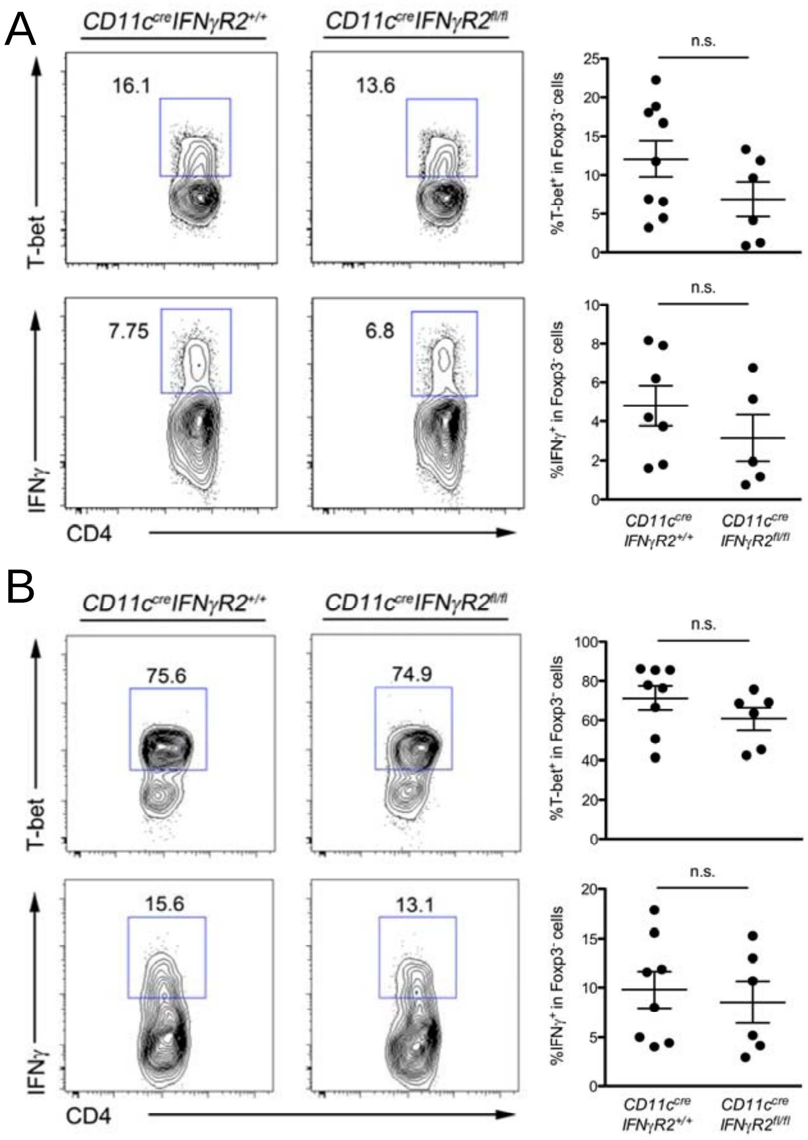
A
CD11c^cre IFNγR2^+/+
CD11c^cre IFNγR2^fl/fl
16.1
13.6
T-bet
7.75
6.8
IFNγ
CD4
n.s.
%T-bet+ in Foxp3- cells
%IFNγ+ in Foxp3- cells
B
75.6
74.9
15.6
13.1

Figure S4.

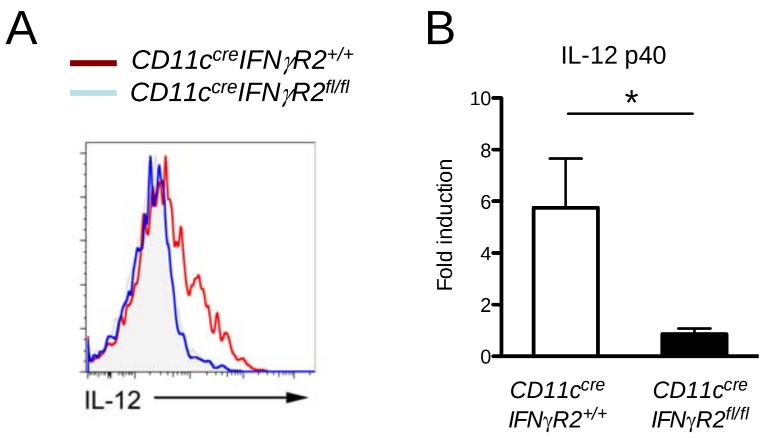

A
CD11c^cre IFNγR2^+/+
CD11c^cre IFNγR2^fl/fl
IL-12
B
IL-12 p40
*
Fold induction
10
8
6
4
2
0
CD11c^cre IFNγR2^+/+
CD11c^cre IFNγR2^fl/fl

Figure S5.

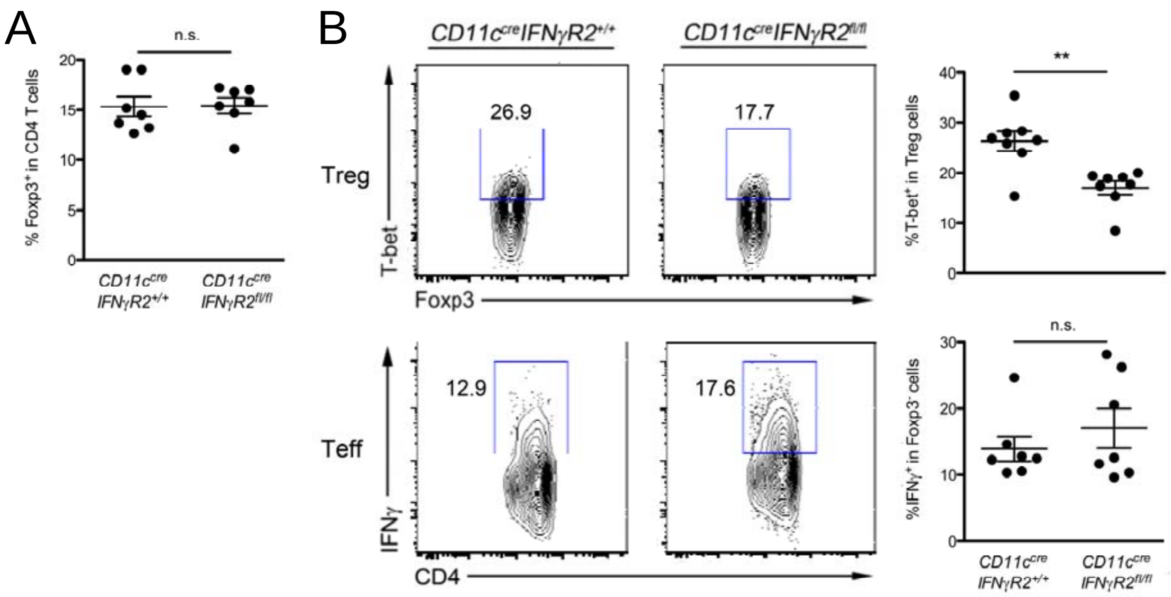
A
n.s.
% Foxp3+ in CD4 T cells
20
15
10
5
0
CD11c^cre IFNγR2^+/+
CD11c^cre IFNγR2^fl/fl
B
CD11c^cre IFNγR2^+/+
CD11c^cre IFNγR2^fl/fl
Treg
26.9
17.7
T-bet
Foxp3
**
%T-bet+ in Treg cells
40
30
20
10
0
Teff
12.9
17.6
IFNγ
CD4
n.s.
%IFNγ+ in Foxp3- cells
30
20
10
0
CD11c^cre IFNγR2^+/+
CD11c^cre IFNγR2^fl/fl

Figure S6.

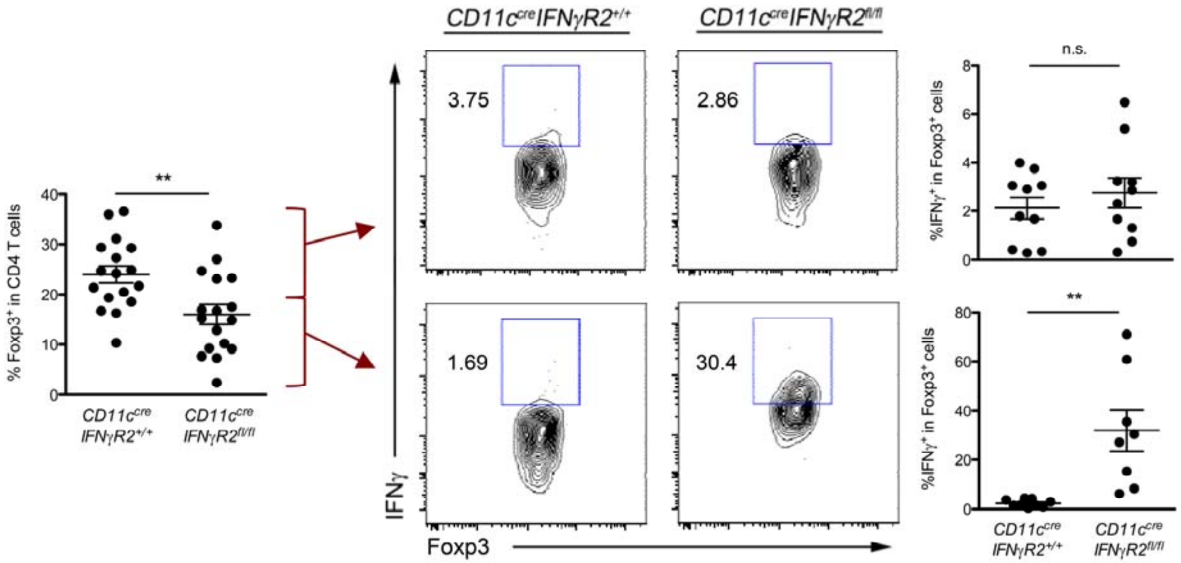
CD11c^cre IFNγR2^+/+
CD11c^cre IFNγR2^fl/fl
% Foxp3+ in CD4 T cells
**
3.75
2.86
1.69
30.4
IFNγ
Foxp3
%IFNγ+ in Foxp3+ cells
n.s.
**
CD11c^cre IFNγR2^+/+
CD11c^cre IFNγR2^fl/fl

Figure S7.

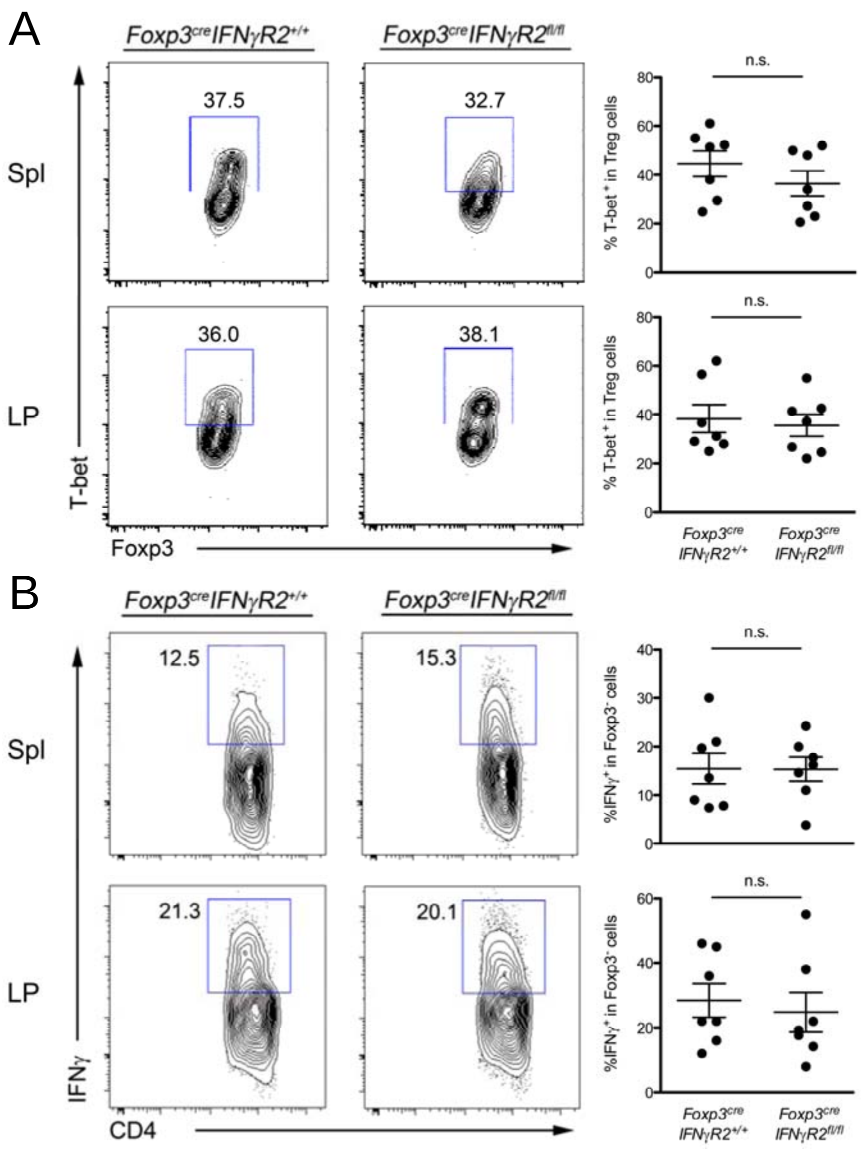
A
Foxp3creIFNγR2+/+
Foxp3creIFNγR2fl/fl
Spl
LP
37.5
32.7
36.0
38.1
T-bet
Foxp3
% T-bet+ in Treg cells
n.s.
B
12.5
15.3
21.3
20.1
IFNγ
CD4
%IFNγ+ in Foxp3- cells
Foxp3cre IFNγR2+/+
Foxp3cre IFNγR2fl/fl

Figure S8.

A

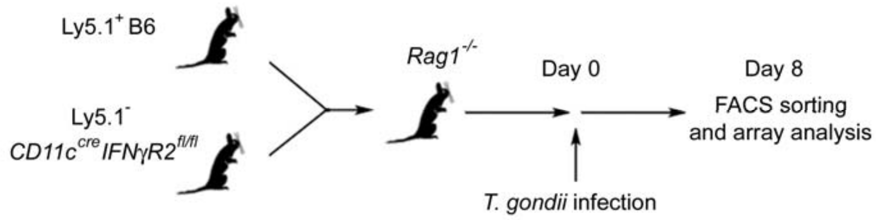

B C

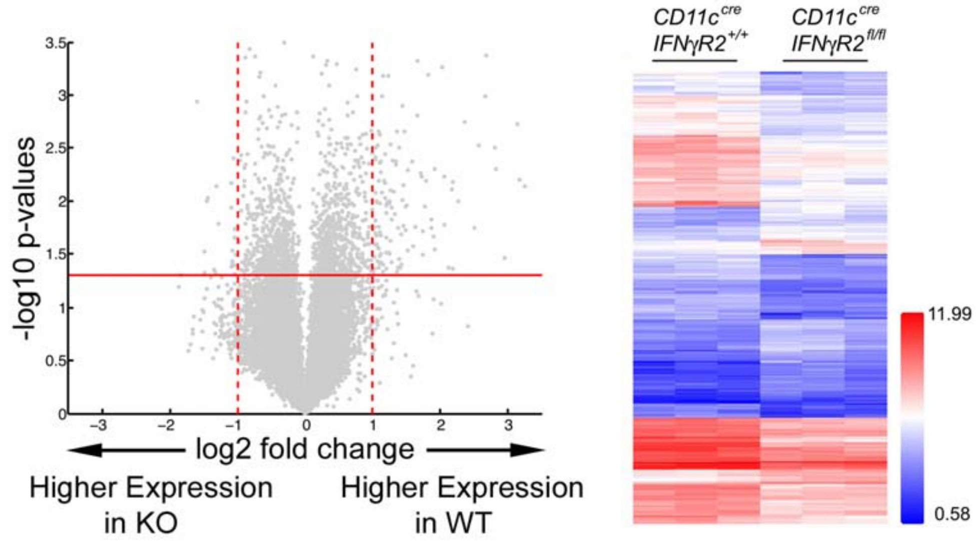

D

Gene Symbol	Fold Change	p-value
Gbp1	8.46	0.000579
Igkv4-91	7.93	0.00688
Gpr33	7.52	0.000254
Gbp4	6.84	0.000176
Cd5l	6.78	0.031278
Abca13	6.55	0.00001
Chi3l1	6.25	0.001099
Iigp1	6.17	0.000394
Gbp11	5.86	0.002114
Fpr1	5.2	0.029251
Igtp	5.01	0.00023
Gbp10	4.9	0.00009
Gbp2	4.88	0.00007
Fcgr4	4.78	0.014924
Tgtp2	4.6	0.002558
Serpina3f	4.59	0.001182
Hk3	4.4	0.000308
Ctse	4.34	0.001942
Smpdl3b	4.21	0.002381
Pydc3	4.15	0.017209

Gene Symbol	Fold Change	p-value
Speer7-ps1	-4.1	0.033368
Olfr134	-4.02	0.044826
Olfr192	-3.46	0.036738
Olfr1239	-3.41	0.045242
Klra9	-3.25	0.033378
Sprr2f	-3.22	0.027016
Scn3a	-3.2	0.008487
Hist1h2bn	-3.1	0.013495
Cd209a	-3.02	0.005273
Plcb4	-2.89	0.000658
Dapk1	-2.8	0.010284
Gabbr1	-2.77	0.00058
Ccl17	-2.62	0.024877
Olfr555	-2.61	0.038238
Treml2	-2.59	0.034439
Olfr1260	-2.5	0.040934
Olfr58	-2.5	0.018341
Mmp12	-2.49	0.000046
Vmn2r86	-2.28	0.025986
Serpinb9e	-2.28	0.046159

Figure S9.

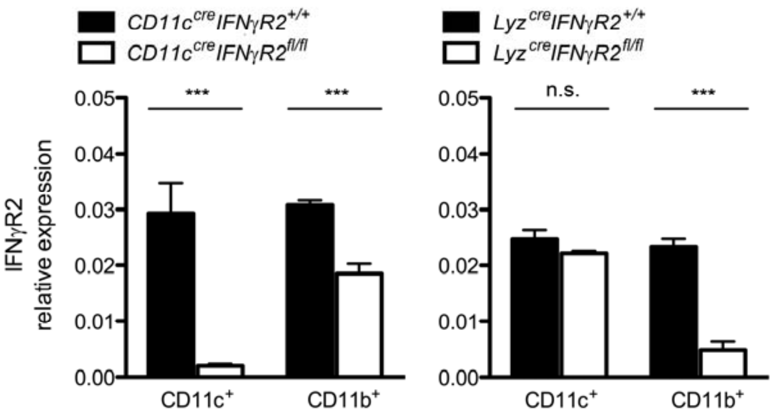

CD11c^{cre}IFNγR2$^{+/+}$
CD11c^{cre}IFNγR2$^{fl/fl}$
Lyz^{cre}IFNγR2$^{+/+}$
Lyz^{cre}IFNγR2$^{fl/fl}$
IFNγR2
relative expression
0.05
0.04
0.03
0.02
0.01
0.00
***
n.s.
CD11c^{+}
CD11b^{+}

Figure S10.

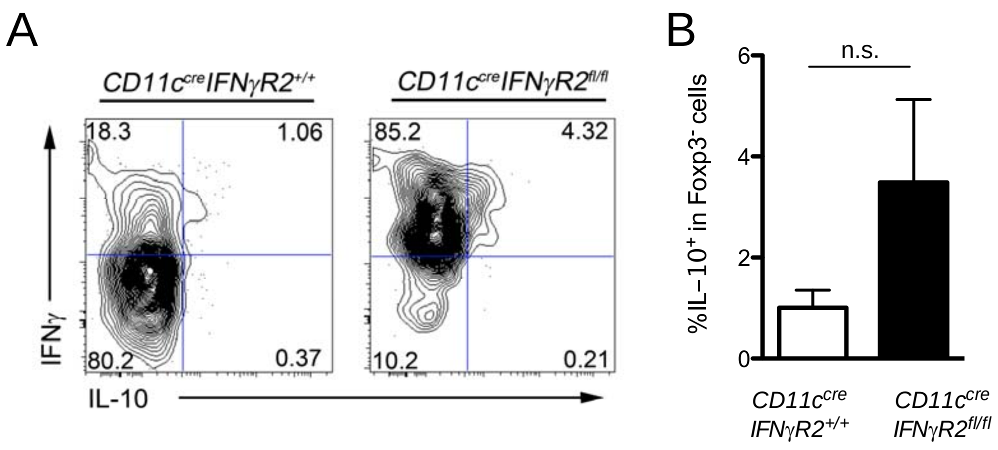
A
CD11c^cre IFNγR2^+/+
CD11c^cre IFNγR2^fl/fl
18.3
1.06
80.2
0.37
85.2
4.32
10.2
0.21
IFNγ
IL-10
B
n.s.
6
4
2
0
%IL−10+ in Foxp3- cells
CD11c^cre IFNγR2^+/+
CD11c^cre IFNγR2^fl/fl

Figure S11.

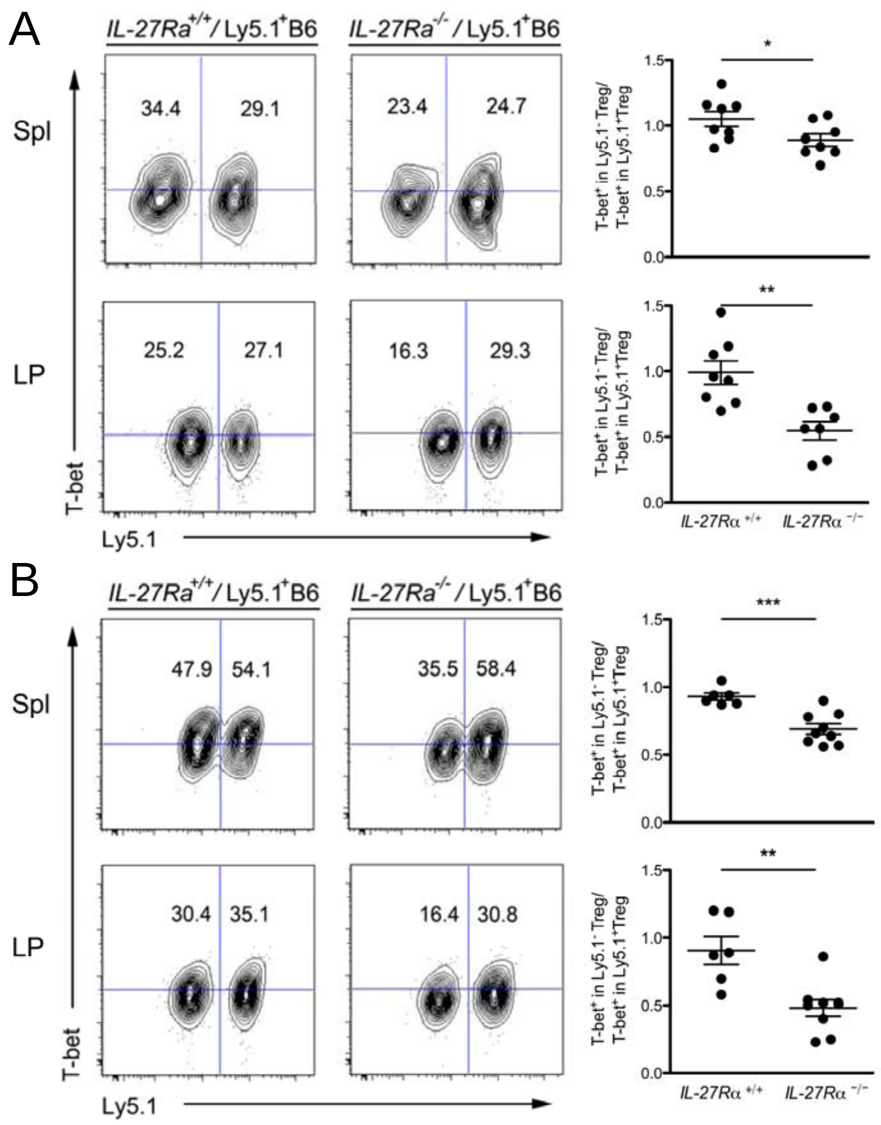
A
IL-27Ra+/+/ Ly5.1+B6
IL-27Ra-/- / Ly5.1+B6
Spl
LP
34.4
29.1
23.4
24.7
25.2
27.1
16.3
29.3
T-bet
Ly5.1
T-bet+ in Ly5.1- Treg/
T-bet+ in Ly5.1+Treg
*
**
1.5
1.0
0.5
0.0
IL-27Rα +/+
IL-27Rα −/−
B
IL-27Ra+/+/ Ly5.1+B6
IL-27Ra-/- / Ly5.1+B6
Spl
LP
47.9
54.1
35.5
58.4
30.4
35.1
16.4
30.8
T-bet
Ly5.1
T-bet+ in Ly5.1- Treg/
T-bet+ in Ly5.1+Treg
***
**
1.5
1.0
0.5
0.0
IL-27Rα +/+
IL-27Rα −/−
